# Nebulized nitroglycerin as an adjuvant drug in management of persistent pulmonary hypertension of newborns: a randomized controlled trial

**DOI:** 10.1007/s00431-025-06381-5

**Published:** 2025-09-01

**Authors:** Marwa Mohammed Farag, Hesham Abd El-Rahim Ghazal, Aly Mohamed Abdel-Mohsen, Moataz Ahmed Rezk

**Affiliations:** Pediatric Department, Alexandria University Hospital, Alexandria, Egypt

**Keywords:** Tissue Doppler MPI, Oxygen saturation index, Pulmonary vasodilators, Nebulized nitroglycerine, RVO, LVO

## Abstract

The objective was to evaluate the effect of nebulized nitroglycerin (NNG) in neonates with persistent pulmonary hypertension (PPHN). The study focused on assessing changes in echocardiographic and clinical parameters following its administration. This is a randomized controlled trial that included 80 full-term newborns diagnosed with PPHN within 72 h after birth. Participants were randomized into two groups: the NNG group received nebulized nitroglycerin, while the control group did not receive it as adjunctive therapy to PPHN. Clinical parameters and echocardiographic measures were evaluated before intervention, and subsequently at 24 and 48 h. The primary outcome was the estimated systolic pulmonary artery pressure in both groups. Comparative analyses were conducted between the groups, along with repeated measures within each group. The NNG group exhibited a significant improvement in the oxygenation index and oxygen saturation index (OI&OSI) on days 2 and 3. There was a marked reduction in the systolic pulmonary artery pressure/systolic systemic pressure (SPAP/SSP) ratio, accompanied by a significant enhancement in biventricular systolic function and cardiac output. The myocardial performance index (MPI), as measured by tissue-Doppler echocardiography, was notably decreased in the NNG group on day 3. Furthermore, the requirements for ventilatory support and inotropic agents were significantly lower in the NNG group. Survival analysis indicated a more favorable weaning course in the NNG group.

*Conclusion*: NNG was effective in managing neonates with PPHN and might be a promising, alternative therapy for PPHN, especially in settings where inhaled nitric oxide is unavailable.

*Trial registration*: Clinical-Trial.gov identifier: NCT05741229 and was registered on February 14, 2023. URL: https://clinicaltrials.gov/study/NCT05741229.

**Supplementary Information:**

The online version contains supplementary material available at 10.1007/s00431-025-06381-5.

## Introduction

Pulmonary hypertension of the newborn (PPHN) is a life-threatening condition characterized by the failure of the pulmonary vasculature to undergo the normal decrease in resistance after birth. It leads to sustained high pulmonary pressures, right-to-left extrapulmonary shunting, and severe systemic hypoxemia, hallmark features of PPHN. This syndrome is a significant cause of morbidity and mortality in NICUs, particularly among full-term and late preterm neonates [1–3].

The clinical presentation of PPHN is often subtle but can escalate rapidly. Affected neonates may exhibit cyanosis refractory to oxygen therapy, labile oxygen saturations, tachypnea, and signs of respiratory distress. Diagnosis is primarily clinical but is confirmed and assessed in severity by echocardiography, which demonstrates elevated pulmonary pressures, right-to-left shunting through the patent ductus arteriosus or foramen ovale, and right ventricular hypertrophy. Early recognition and management are crucial to improving outcomes [4, 5].

Therapeutic goals in PPHN focus on optimizing oxygenation, promoting pulmonary vasodilation, supporting myocardial function, and minimizing oxygen toxicity. Conventional therapies include supplemental oxygen, mechanical ventilation strategies aimed at lung recruitment, and the use of vasodilators. Inhaled nitric oxide (iNO) is the standard of care for selective pulmonary vasodilation. However, its use is limited in many resource-constrained settings due to high costs and the need for specialized equipment. Alternative therapies are thus critically needed, especially in regions where iNO and extracorporeal membrane oxygenation (ECMO) are not available [6–8].

Nitroglycerin, a nitric oxide donor, has emerged as a potential alternative agent for pulmonary vasodilation. Administered via inhalation, nitroglycerin acts directly on the pulmonary vasculature, promoting selective vasodilation while minimizing systemic hypotension. Previous studies in pediatric patients with pulmonary hypertension secondary to congenital heart disease and in adults undergoing cardiac surgeries have demonstrated the safety and efficacy of nebulized nitroglycerin (NNG). Its rapid onset of action, ease of administration through standard nebulizers, and low cost make it an attractive therapeutic option in neonates with PPHN [9–11].

Despite the theoretical benefits and preliminary clinical observations supporting the use of NNG, robust clinical evidence in neonates with PPHN remains scarce. Most existing studies are limited by small sample sizes, heterogeneous populations, or observational designs. Well-designed randomized controlled trials are necessary to establish the efficacy and safety of NNG in this vulnerable population. Furthermore, evaluation through objective echocardiographic parameters and clinical indicators is essential to comprehensively assess the impact of such therapy [12, 13].

Hence, the current study aimed to investigate the effect of NNG as an adjunctive treatment in full-term neonates with PPHN. By evaluating echocardiographic parameters such as pulmonary artery pressure, ventricular function, and ductal shunting, alongside clinical indicators of oxygenation and ventilation needs, this study seeks to provide evidence-based insights into the role of NNG in improving outcomes for neonates suffering from this critical condition.

## Methods

### Study design, participants, eligibility criteria, and settings

This research was structured as a randomized controlled clinical trial and took place at the Neonatal Intensive Care Unit (NICU) of Alexandria University Maternity Hospital (AUMH) from January 2024 to December 2024. AUMH, a tertiary care facility located on Egypt’s Northern coast, serves four governorates. The study was prospectively registered in the ClinicalTrials.gov database (NCT05741229). The study was conducted according to current Consolidated Standards of Reporting Trials (CONSORT) reporting guidelines.

The study adhered to the ethical standards of the institutional research committee and the 1964 Helsinki Declaration, including its subsequent amendments. Approval for the trial was granted by the ethics committee of Alexandria University on 17/11/2022, with approval number 0201737, IRB no 00012098, and FWA no. 00018699.

**Fig. 1:**
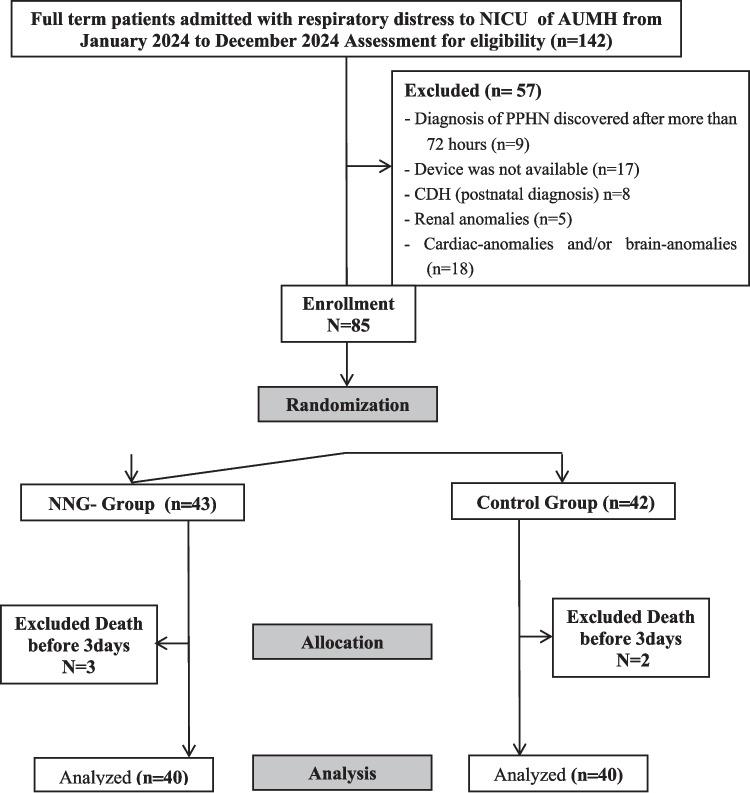
Consort flowchart of studied patients

The study included 80 full-term newborns diagnosed with PPHN within the first 72 h of life, with informed consent obtained from their parents, consort flow chart was provided in Fig. 1. Eligible participants were those born at or after 37 weeks of gestation, requiring high levels of inspired oxygen (FiO₂ ≥ 50%) despite lung recruitment efforts or showing echocardiographic signs of PPHN, such as a high TR gradient > 50 mmHg and an abnormal D-shaped LV configuration. Exclusion criteria were a PPHN diagnosis beyond 72 h of life and the presence of major congenital anomalies (Fig. 1).

### Randomization and blinding

Randomization was conducted by assigning participants to two groups in a 1:1 ratio using random permuted blocks of sizes 4 and 6, prepared by an independent researcher using computer-generated random numbers. Neonatology residents allocated participants to the groups using serially numbered cards. Patients remained in their assigned group for the duration of the intervention, with no crossover allowed. Blinding was not feasible due to the nature of the interventions.

### Intervention

Informed consent was obtained from the guardians of the patients upon NICU admission. Patients were assigned to two groups: group1 (NNG group) and group2 (control group). Patients in the NNG group received standard care of PPHN, along with nebulized nitroglycerin at a dose of 2.5 μg/kg/min for 10 m, repeated every 4 h. NNG was prepared by diluting the original solution to a final concentration of 250 μg/ml, with dosing adjusted according to patient weight. Delivery was carried out using an Aergeon Jet nebulizer during ventilation. Both the NNG and control groups received conventional PPHN treatments, including oxygenation, ventilation, enteral sildenafil, and inotropic support. Also, patients were monitored regarding possible side effects of NNG like hypotension.

### Primary and secondary outcomes

Primary outcomes focused on improvements in estimated systolic pulmonary artery pressure (SPAP) in the two groups. Secondary outcomes included other echocardiographic parameters and oxygenation indices, the duration of mechanical ventilation, length of NICU stay, need for additional inotropic support, and overall mortality.

### Clinical and echocardiographic assessments

Clinical assessment involved continuous monitoring of heart rate, blood pressure, oxygen saturation, respiratory parameters, mean airway pressure (MAP), and ventilatory settings. Blood gas analyses and laboratory tests were conducted periodically to monitor systemic oxygenation and detect any metabolic disturbances. Oxygen saturation and FIO2 were recorded, and the oxygen saturation index (OSI) was calculated at the time of scans. Oxygenation index (OI) was calculated according to equation, reported by Muniraman et al. [14]

Echocardiographic assessments were performed three times for each patient: the first scan on the first day before intervention, and the second and third scans on days 2 and 3, respectively, after the intervention. Initial echocardiographic evaluations were conducted to assess the ductus arteriosus (DA), estimate SPAP by measuring the tricuspid regurgitation (TR) gradient, evaluate the functions of both the right and left ventricles, and rule out any structural congenital heart defects. SPAP was corrected by calculating sPAP/SSP, according to Piastra et al. [15]. A single operator performed the imaging using a GE Vivid iq premium machine equipped with a GE 12S-RS probe, operating within a frequency range of 5–11 MHz. The operator utilized 2D, M-mode, color Doppler, continuous wave, and pulsed wave (PW) Dopplers. Doppler volumetric assessments of the superior vena cava flow (SVCF), left ventricular output (LVO), and right ventricular output (RVO) were conducted following the Evans and Kluckow methodology [16–20].

The velocity time integral (VTI) was derived from the Doppler velocity tracings and averaged over five consecutive cardiac cycles for SVCF and three consecutive cycles for RVO and LVO estimations. Heart rate was determined from the peak-to-peak intervals of the Doppler velocity time signals. The ductus arteriosus was measured at its narrowest point before entering the main pulmonary artery. The size of the DA and the direction of shunts were evaluated, along with the estimated SPAP based on the TR gradient and right atrial pressure. Left ventricular systolic function was assessed using fractional shortening (FS) and ejection fraction (EF), while systolic right ventricular function was evaluated using tricuspid annular plane systolic excursion (TAPSE). Tissue Doppler imaging (TDI) was employed to calculate the right myocardial performance index (MPI). The tissue Doppler method allows for the measurement of the right MPI, as well as é, á, and ś, all from a single image. The isovolumic time (measured as the time between tricuspid valve closure (end of á) and opening (start of é) minus the ejection time) is divided by the ejection time [21].

### Statistical analysis

A minimum total sample size of 34 patients with PPHN of newborn (17 per group) was required to detect a difference of 9 in mean pulmonary arterial blood pressure (MPAP) between a group receiving nebulized nitroglycerin (NNG) and a placebo group [22], with estimated group standard deviations of 8. This was to evaluate the effectiveness of nebulized nitroglycerin on the echocardiographic findings of patients with persistent pulmonary hypertension using an independent *t* test with a significance level of 0.05 and 90% power.

Data analysis was performed using IBM SPSS software version 20.0. The Kolmogorov–Smirnov test was applied to verify the normality of distribution. Qualitative data were described using numbers and percentages, while quantitative data were described using range (minimum and maximum), mean, standard deviation, median, and interquartile range (IQR). The Student *t* test, Monte Carlo test, chi-square test (*χ*^2^), and Fisher exact test were used to compare the two groups regarding different variables. The Friedman test and the Wilcoxon signed-rank test as post hoc analyses were employed for comparison and pairwise evaluations among repeated measures at various timepoints, respectively. A Kaplan–Meier survival plot was created to track the weaning time in the studied groups.

## Results

One hundred forty-two full-term infants with respiratory distress (RD) were evaluated for eligibility, with 57 infants excluded, as shown in Fig. 1. The remaining 85 infants were randomly assigned to either the control group or the NNG group, with 80 infants completing the study and having the three scans. Forty infants were allocated to each group. The demographic profiles, antenatal risk factors, and resuscitation needs were similar between the two groups under study. Likewise, laboratory parameters did not exhibit any statistically significant differences. In addition, time of recruitment of patients was comparable in both groups (Table 1 and eTable (1) in Supplementary information).

**Table 1:** Comparison between the two studied groups according to demographic data

	NNG (***n*** = 40)	Control (***n*** = 40)	***p***
Sex ***n*** (%)			
Male	17 (42.5)	14 (35.0)	0.491
Female	23 (57.5)	26 (65.0)	
Gestational age (weeks)			0.935
Median (IQR)	38.0 (37.0–38.50)	38.0 (37.0–38.0)	
Birth weight (kg)			
Mean ± SD (min.–max.)	2.81 ± 0.49 (2.0–3.90)	2.96 ± 0.55(2.0–4.20)	
Type of delivery, ***n*** (%)			
Vaginal delivery	12 (30)	10 (25)	0.617
C-section	28 (70)	30 (75)	
Antenatal steroids, ***n*** (%)			
Yes	11 (27.5)	13 (32.5)	0.626
No	29 (72.5)	27 (67.5)	
APGAR at 5 min)			0.845
Median (IQR)	7.0 (5.0–8.0)	7.0 (5.0–7.0)	
SNAPPE-II score			0.350
Median (IQR)	36.5(19.50–59.0)	42.0 (24.0–82.0)	
Postnatal age at the recruitment (hours)			0.870
Median (IQR)	15.0 (9.0–27.50)	15.50 (8.0–30.0)	

*SNAPPE* Score for Neonatal Acute Physiology with Perinatal Extension-II

**Fig. 2:**
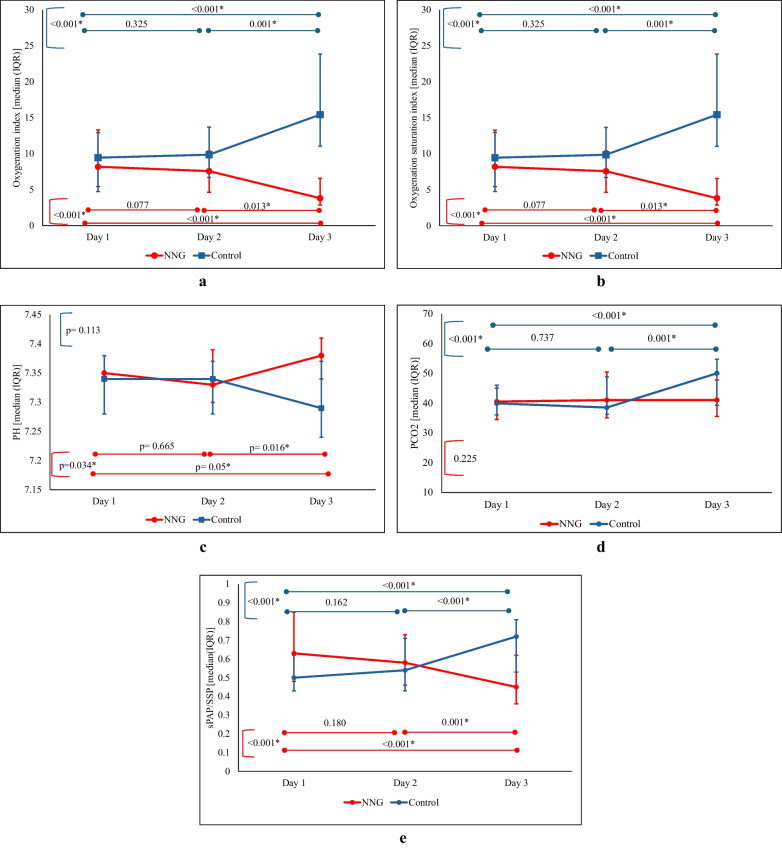
A graphical illustration of oxygenation parameters, including oxygenation index (**a**) and oxygen saturation index (**b**), pH (**c**) and PCO2 (**d**) levels, and SPAP/SSP (**e**) for both groups across three distinct time points

Clinical outcomes indicated that initial heart rate and systolic and diastolic blood pressures were comparable between the groups. However, by the third day, the NNG group had significantly higher systolic blood pressure and mean arterial pressure than the control group, indicating improved cardiovascular stability, eTable (3) in Supplementary information. In the current study, hypotension secondary to NNG was not reported. The NNG group exhibited significantly higher pH values and lower PCO₂ levels, suggesting enhanced gas exchange and respiratory function (eTable (1) in Supplementary information and Fig. 1). By the second and third days, oxygenation parameters, including SPO2, FIO2, OI, and OSI showed marked improvement in the NNG group, indicating better pulmonary function (eTable (1) in Supplementary information and Fig. 2). Following the administration of NNG, both OSI and OI demonstrated significant improvement, whereas in the control group, these parameters deteriorated. Additionally, pH levels showed a marked improvement post-NNG administration, while pCO2 levels significantly worsened in the control group, despite the application of conventional measures.

The mode of ventilation also shifted positively; by the third day, a significantly larger proportion of neonates in the NNG group were supported by less invasive methods like nasal continuous positive airway pressure (NCPAP), while more infants in the control group remained on high-frequency oscillatory ventilation (HFOV) (eTable (1), eFig. (1)). Furthermore, mean airway pressure (MAP) values by the second and third days reflected improved lung compliance and reduced need for ventilatory support (eTable (1) in Supplementary information).

Although the initial estimated SPAP was higher in the NNG group, a significant reduction in SPAP was observed by the third day compared to the control group. Similarly, while the SPAP/SSP ratio was notably elevated in the NNG group on the first day, by the third day, this ratio had shifted to be significantly higher in the control group following the administration of NNG. Furthermore, the ratio demonstrated a marked decrease in the NNG group and an increase in the control group on day 3 compared to day 1, as depicted in Fig. 2 and eTable (2).

Biventricular functions, assessed by TAPSE, EF, and FS, improved significantly among neonates treated with NNG (eFig. (1), Table 2). Further echocardiographic evaluations showed that RVO and LVO, and stroke distances were significantly better in the NNG group by the third day. Right ventricular MPI improved more favorably in the NNG group, indicating enhanced myocardial function. PDA size tended to decrease more in the NNG group, and a favorable shift toward left-to-right shunting was observed, reflecting improved pulmonary vascular resistance (eTable (2) in Supplementary information). Although EF, FS, TAPSE, and RV TD_MPI initially showed no notable differences between the two groups, they exhibited significant improvement in the NNG group compared to the control group. Both RVO and LVO also showed marked improvement in the NNG group, Table 2 and eFig. (1) in Supplementary information.

**Table 2:** Comparison between the two groups regarding echocardiographic parameters in the three scans, primary, and secondary outcomes

	Group		***p***
	NNG	Control	
sPAP/SSP (day 1)			
Median (IQR)	0.63 (0.48–0.85)	0.50 (0.43–0.62)	0.021*
sPAP/SSP (day 2)			
Median (IQR)	0.58 (0.46–0.73)	0.54 (0.43–0.71)	0.541
sPAP/SSP (day 3)			
Median (IQR)	0.45 (0.36–0.62)	0.72 (0.53–0.81)	< 0.001^*^
***p***	< 0.001*	< 0.001*	
Sig. between days	*p*1 = 0.180 *p*2 < 0.001*, *p*3 = 0.001*	*p*1 = 0.162 *p*2 < 0.001*, *p*3 < 0.001*	
Ejection fraction D1 (%)			
Mean ± SD (min.–max.)	69 ± 12(45–88)	67 ± 11(38–86)	0.502
Ejection fraction D2 (%)			
Mean ± SD (min.–max)	69 ± 10(42–88)	68 ± 10(50–89)	0.545
Ejection fraction D3 (%)			
Median (IQR)	72 (62–79)	65 (57–72)	0.005^*^
***p***	0.694	0.061	
Fractional shortening D1 (%)			
Mean ± SD (min.–Max.)	36 ± 9(21–55)	34 ± 8(17–51)	0.418
Fractional shortening D2 (%)			
Median (IQR)	37 (33–41)	34 (30–39)	0.134
Fractional shortening D3 (%)			
Min.–max	20–51	19–49	0.019*
Mean ± SD	37 ± 8	33 ± 7	
Test of Sig	*χ*^2Friedman^ = 0.247	*χ*^2Friedman^ = 4.397	
***p***	0.884	0.111	
SVC flow D1 (ml/min/kg)			
Median (IQR)	108.39 (75.66–137.36)	96.01 (68.53–132.34)	0.488
SVC flow D2 (ml/min/kg)			
Median (IQR)	115.96 (68.53–146.10)	101.68 (67.99–116.45)	0.131
SVC flow D3 (ml/min/kg)			
Median (IQR)	114.26 (81.60–143.67)	102.34 (72.38–141.09)	0.419
***p***	0.975	0.535	
TAPSE D1 (cm)			
Median (IQR)	0.71 (0.60–0.80)	0.75 (0.53–0.90)	0.715
TAPSE D2 (cm)			
Median (IQR)	0.80 (0.70–0.90)	0.70 (0.60–0.90)	0.239
TAPSE D3 (cm)			
Median (IQR)	0.90 (0.76–1.08)	0.70 (0.50–0.70)	< 0.001*
***p***	< 0.001*	< 0.001*	
Sig. between days	*p*1 = 0.05* *p*2 < 0.001*, *p*3 < 0.001*	*p*1 = 0.576 *p*2 < 0.001*, *p*3 = 0.001*	
RVO D1 (ml/min/kg)			
Median (IQR)	206 (128.5–279.25)	229 (146.75–323.25)	0.356
RVO D2 (ml/min/kg)			
Median (IQR)	234.5 (195.25–320.5)	210 (168.25–334.25)	0.450
RVO D3 (ml/min/kg)			
Median (IQR)	304 (226–382.5)	189.5 (150.25–250.5)	0.001*
***p***	< 0.001*	0.139	
Sig. between days	*p*1 = 0.003* *p*2 < 0.001*, *p*3 = 0.001*		
TD-MPI RV D1			
Min.–max	0.35–0.89	0.37–0.87	0.130
Mean ± SD	0.63 ± 0.12	0.59 ± 0.13	
TD-MPI RV D2			
Min.–max	0.41–0.81	0.33–0.87	0.435
Mean ± SD	0.61 ± 0.11	0.63 ± 0.11	
TD-MPI RV D3			
Median (IQR)	0.52 (0.44–0.62)	0.60 (0.56–0.73)	0.003*
***p***	0.002*	< 0.001*	
Sig. between days	*p*1 = 0.118 *p*2 = 0.001*, *p*3 = 0.057	*p*1 = 0.012* *p*2 < 0.001*, *p*3 = 0.180	
LVO D1(ml/min/kg)			
Median (IQR)	110 (88.25–147.25)	102.5 (77.25–143.5)	0.358
LVO D2 (ml/min/kg)			
Median (IQR)	123.5 (102–172)	98 (68–144.5)	0.009^*^
LVO D3(ml/min/kg)			
Median (IQR)	131.5 (112.25–195.5)	95 (66.25–140.25)	0.001*
***p***	0.014*	0.465	
Sig. between days	*p*1 = 0.199 *p*2 = 0.004*, *p*3 = 0.105		

sPAP/SSP systolic pulmonary artery pressure/systemic systolic pressure, SVC superior vena cava, RVO right ventricular output, LVO left ventricular output, TD-MPI-RV tissue Doppler measured myocardial performance index of right ventricle, TAPSE tricuspid annular plane systolic excursion

^*^Statistically significant at *p*** ≤ 0.05**

**Fig. 3:**
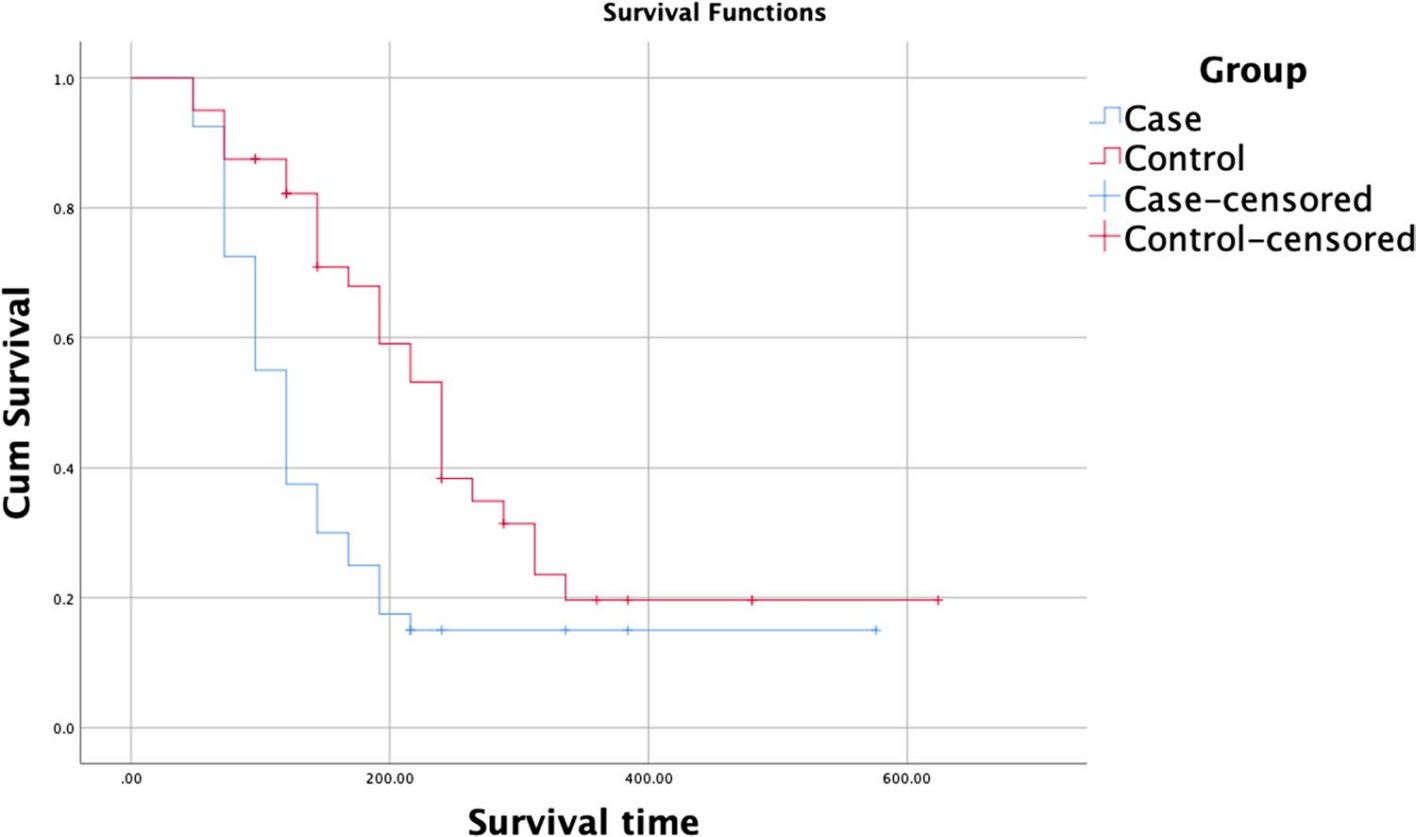
Kaplan–Meier survival plot representing time course of weaning from ventilatory support (terminal events) and occurrence of death (censored cases) in the two studied groups, NNG (case) and control groups

Nevertheless, the need for inotropic support significantly decreased in the NNG group by the second and third days, highlighting improved hemodynamic stability, eTable (4). The causes of PPHN in both studied groups were demonstrated in eTable (5), with no significant differences between the studied groups. The duration of mechanical ventilation and NICU stay was shorter among neonates receiving nebulized nitroglycerin, eTable (6), although mortality differences were not statistically significant. Kaplan–Meier survival analysis in Fig. 3 and eTable (7) demonstrates earlier weaning from mechanical ventilation in NNG group. While eFig. (1) and eTable (8) demonstrate time-linked mortality of both groups using Kaplan–Meier survival analysis.

## Discussion

PPHN remains a major cause of neonatal morbidity and mortality, resulting from the failure of the pulmonary circulation to adapt after birth. The elevated pulmonary vascular resistance leads to right-to-left shunting through PDA and foramen ovale, causing severe hypoxemia. Despite advances in neonatal intensive care, effective management remains challenging, especially in low-resource settings where access to iNO and ECMO is limited. As a result, there has been growing interest in alternative therapies that are both accessible and effective, such as NNG, which acts as a nitric oxide donor promoting selective pulmonary vasodilation without significant systemic hypotension [13].

Fortas and colleagues provided a proposal for administering pulmonary vasodilators in cases of resistant PPHN. Their approach relies on the synergistic effects of the drugs, with a preference for nebulization, an understanding of the pathophysiological mechanisms of PPHN, clinical experience with various medications, and the potential presence of specific hemodynamic conditions [23]. The algorithm did not include sildenafil, as previously reported that IV sildenafil added no value to iNO in infants with PPHN [24].

In light of these challenges, our research sought to assess the effectiveness of NNG as a supplementary treatment for managing PPHN. Our results indicated that incorporating NNG into standard therapy notably enhanced both clinical and echocardiographic outcomes compared to conventional treatment alone.

Nitro-glycerine relaxes vascular smooth muscle by intracellular conversion to nitrite ions and then nitric oxide (NO), which in turn activates guanylate cyclase and increases intracellular cGMP, which ultimately leads to phosphorylation of myosin light chain and smooth muscle relaxation. Similarly, sildenafil is a phosphodiesterase 5 inhibitor, therefore causing cGMP accumulation and smooth muscle relaxation. It is quite clear that both sildenafil and nitro-glycerine act on the same pathway and have the same second messenger, hence no expected synergy. While the synergy is well known for medications acting on different pathways. [23, 25] Therefore, sildenafil is a suboptimal drug for the treatment of PPHN and can be accepted only in case of NO unavailability, like the current settings. Also, the efficacy of sildenafil could have been hampered by enteral administration.

The initial characteristics of both groups were well-matched, ensuring proper randomization and reducing bias. Laboratory results were also comparable, confirming that the clinical improvements observed were due to the intervention rather than recovery of underlying sepsis or inflammation and/or anaemia. [26] Regarding hemodynamic parameters, we noted a significant enhancement in systolic blood pressure and stabilization of mean blood pressure in the NNG group by the third day. These findings align with Goyal et al. [27] who found that iNO significantly lowered PAP without inducing systemic hypotension in children with severe PPHN. Similarly, Fikry et al. [28] observed a reduction in pulmonary vascular resistance and right ventricular workload with inhaled NNG, although they mentioned its shorter duration of action compared to milrinone. In contrast, our neonates exhibited sustained improvements over 3 days, possibly due to repeated dosing and differences in neonatal physiology.

Another significant finding was the improvement in the blood gas parameters. By the third day, the NNG group exhibited notably higher arterial pH and lower PaCO₂ levels than the control group, suggesting improved ventilation-perfusion matching. These findings are corroborated by Thunberg et al. [29], who reviewed the application of inhaled nitroglycerin in perioperative pulmonary hypertension and confirmed its capacity to selectively enhance gas exchange without impacting systemic hemodynamics. Furthermore, the improved oxygenation parameters, including a lower oxygen saturation index (OSI) and reduced FiO₂ requirements in the NNG group, align with the observations of Rawat et al. [30], who emphasized the prognostic significance of OSI in neonatal hypoxic respiratory failure. The improvement in the ventilatory support requirements was particularly noteworthy. Analysis of repeated measures within each group revealed that the NNG group demonstrated significant improvement in pH and more stable PaCO₂ levels than the control group. Moreover, OSI and OI markedly increased in the control group and notably decreased in the NNG group.

By the third day, neonates in the NNG group were much more likely to be weaned from HFOV and required lower PEEP and MAP. These findings are in line with the study by Ignarro [31] who described how nitrovasodilators can enhance pulmonary compliance and reduce ventilator dependence by selectively decreasing pulmonary vascular resistance.

In terms of echocardiographic findings, pulmonary artery pressures were significantly lower, and right ventricular function, assessed by TAPSE, was notably better in the NNG group by day three. De Boode et al. [32] and Koestenberger et al. [33] previously reported that pulmonary vasodilators can significantly enhance right ventricular function in neonates, supporting our observations. Furthermore, Singh et al. [34] demonstrated that inhaled nitroglycerin and milrinone exhibit comparable efficacy in reducing pulmonary pressure, thereby reinforcing the validity of our findings regarding the benefits of NNG without significant systemic side effects. On day 3, both RVO and LVO were significantly elevated in the NNG group compared to the control group, and they increased significantly through serial measurements only in the NNG group, indicating enhanced systemic perfusion and improved myocardial performance. The PDA size was reduced and shifted toward a left-to-right shunt in the NNG group, supporting a successful transition to normal postnatal circulation. These findings align with those of Gien et al. [35], who emphasized the importance of reversing ductal shunting as a key therapeutic goal in managing PPHN.

Finally, ventricular function parameters, including EF, FS, and right ventricular MPI, significantly improved in the NNG group. Nevertheless, serial measurements within each group did not reveal any advantage of NNG treatment over the control. Georgiopoulou et al. [36] highlighted the close link between pulmonary hypertension and biventricular dysfunction, and our findings support the notion that effective pulmonary vasodilation can positively influence ventricular performance. Furthermore, the marked reduction in the need for inotropic support among neonates in the NNG group underscores the clinical efficacy of NNG. Mandal et al. [37] corroborated the pulmonary selectivity of inhaled nitroglycerin in their study, which showed a significant reduction in pulmonary pressures without inducing systemic hypotension, aligning with our neonatal data.

### Limitations of the study

The study was conducted at a single center, which may limit the generalizability of the results to other healthcare settings, especially those with different levels of neonatal care. The echocardiographic follow-up period was limited to three days; therefore, the long-term outcomes could not be assessed. The study did not include a comparison with iNO, which remains the gold standard in high-resource settings. Direct comparison would provide stronger evidence regarding the efficacy of NNG.

## Conclusion

In conclusion, our findings suggest that NNG is a promising adjunctive therapy for PHN, offering significant improvements in pulmonary hemodynamics, oxygenation, and cardiac function without systemic side effects. However, further multicenter studies with larger sample sizes and longer follow-up periods are needed to confirm these encouraging results and potentially integrate NNG into standard PPHN management protocols, particularly in resource-limited settings.

## Supplementary Information

Below is the link to the electronic supplementary material.

Supplementary file 1 (DOCX 78.1 KB)

Supplementary file 2 (DOCX 877 KB)

Supplementary file 3 (DOCX 32.2 KB)

## Data Availability

No datasets were generated or analysed during the current study.

## Abbreviations

DBP: Diastolic blood pressure
EF: Ejection fraction
FIO2: Fraction of inspired oxygen
FS: Fractional shortening
HFOV: High frequency oscillatory ventilation
LVO: Left ventricular output
MABP: Mean arterial blood pressure
MAP: Mean airway pressure
MPI: Performance index SVCF superior vena cava flow
NCPAP: Nasal continuous positive airway pressure
NNG: Nebulized nitro-glycerine
OSI: Oxygenation saturation index
O: Oxygenation index
PDA: Patent ductus arteriosus
PFO: Patent foramen ovale
PPHN: Persistent pulmonary hypertension
PTV: Patients triggered ventilation
RD: Respiratory distress
RVO: Right ventricular output
SBP: Systolic blood pressure
sPAP/SSP: Ratio of systolic pulmonary artery pressure and systolic systemic pressure
TAPSE: Tricuspid annular plane systolic excursion
TDI: Tissue Doppler imaging
TR: Tricuspid gradient
