# Supplementary material for: Nebulized nitroglycerin as an adjuvant drug in management of persistent pulmonary hypertension of newborns: a randomized controlled trial
Source: Eur J Pediatr. 2025 Sep 1;184(9):586. doi: 10.1007/s00431-025-06381-5 (PMC12402028; doi:10.1007/s00431-025-06381-5)
Supplement: Supplementary file 1 — (DOCX 78.1 KB) [file 431_2025_6381_MOESM1_ESM.docx]

**s-Table (1): Comparison between the two groups as regards demographic, antenatal and resuscitation data**

|  | | **Group** | | **Test of sig.** | **P** |
| --- | --- | --- | --- | --- | --- |
|  |  | **Case** | **Control** |  |  |
| **Resuscitation** | Routine care | 12 (30.0%) | 8 (20.0%) |  |  |
|  | Initial steps | 13 (32.5%) | 10 (25.0%) | χ^2^ = 3.125 | 0.373 |
|  | PPV | 12 (30.0%) | 15 (37.5%) |  |  |
|  | ETT | 3 (7.5%) | 7 (17.5%) |  |  |
| **Antenatal risk factors** | |  |  |  |  |
| Anemia | | 8 (20.0%) | 9 (22.5%) | χ^2^ = 0.075 | 0.785 |
| Infection | | 3 (7.5%) | 8 (20.0%) | χ^2^ = 2.635 | 0.105 |
| DM | | 10 (25.0%) | 8 (20.0%) | χ^2^ = 0.287 | 0.592 |
| PET | | 8 (20.0%) | 9 (22.5%) | χ^2^ = 0.075 | 0.785 |
| GHT | | 8 (20.0%) | 4 (10.0%) | χ^2^ = 1.569 | 0.210 |

p: p-value for comparing between the **two studied groups**

χ^2^: Chi-square test MOD: Mode of delivery GA: gestational age BWT: birth weight DM: Diabetes mellitus PET: Preeclampsia

GHT: gestational hypertension

**S-Table (2): Comparison between the two groups as regards laboratory and microbiological data**

|  | | **Group** | | **Test of sig.** | **P** |
| --- | --- | --- | --- | --- | --- |
|  |  | **Case** | **Control** |  |  |
| **Hb (g/dl)** | |  |  |  |  |
| Min. – Max. | | 10.5 – 18.3 | 12.5 – 22.6 | U= 729.5 | 0.497 |
| Mean ± SD. | | 15.0 ± 1.8 | 15.6 ± 2.1 |  |  |
| Median (IQR) | | 15.1 (14.1 – 16.4) | 15.4 (14.2 – 16.5) |  |  |
| **WBCs count X103/dl** | |  |  |  |  |
| Min. – Max. | | 4.1 – 36.4 | 3.4 – 39.0 | U= 718.5 | 0.433 |
| Mean ± SD. | | 14.2 ± 6.5 | 15.4 ± 7.0 |  |  |
| Median (IQR) | | 13.2 (9.8 – 17.3) | 13.8 (11.6 – 20.0) |  |  |
| **Platelets X103/dl** | |  |  |  |  |
| Min. – Max. | | 137.0 – 461.0 | 79.0 – 394.0 | t= 1.140 | 0.258 |
| Mean ± SD. | | 250.3 ± 78.0 | 230.7 ± 75.4 |  |  |
| Median (IQR) | | 233.0 (197.0 – 300.0) | 231.0 (164.0 – 298.0) |  |  |
| **CRP Mg/L** | |  |  |  |  |
| Min. – Max. | | 0.1 – 67.0 | 0.1 – 25.0 | U= 705.5 | 0.363 |
| Mean ± SD. | | 7.4 ± 14.0 | 6.9 ± 6.8 |  |  |
| Median (IQR) | | 3.0 (1.5 – 6.3) | 3.3 (2.2 – 11.9) |  |  |
| **Blood Cultures** | Positive | 8 (20.0%) | 15 (37.5%) | χ^2^ = 2.990 | 0.084 |
|  | Negative | 32 (80.0%) | 25 (62.5%) |  |  |

U: Mann Whitney test t: Independent sample t test χ^2^: Chi-square test p: p-value for comparing between the **two studied groups**

**S-Table (3): Comparison between the two groups as regards vital signs at time of scans**

|  | **Group** | | **Test of sig.** | **P** |
| --- | --- | --- | --- | --- |
|  | **NNG** | **Control** |  |  |
| **HR B/min (day 1)** |  |  |  |  |
| Min. – Max. | 100.0 – 197.0 | 110.0 – 200.0 | U= 721 | 0.447 |
| Mean ± SD. | 149.4 ± 21.2 | 145.0 ± 18.1 |  |  |
| Median (IQR) | 145.5 (138.5 – 156.5) | 144.0 (136.0 – 150.0) |  |  |
| **HR B/min (day 2)** |  |  |  |  |
| Min. – Max. | 115.0 – 193.0 | 115.0 – 206.0 | U= 763 | 0.722 |
| Mean ± SD. | 148.6 ± 18.1 | 147.3 ± 17.4 |  |  |
| Median (IQR) | 148.5 (139.0 – 162.0) | 148.0 (140.5 – 155.0) |  |  |
| **HR B/min (day 3)** |  |  |  |  |
| Min. – Max. | 110.0 – 195.0 | 105.0 – 197.0 | t= -0.709 | 0.481 |
| Mean ± SD. | 147.8 ± 18.7 | 150.9 ± 20.1 |  |  |
| Median (IQR) | 145.0 (135.5 – 159.0) | 150.0 (138.5 – 158.0) |  |  |
| **SBP mmHg (day 1)** |  |  |  |  |
| Min. – Max. | 54.0 – 128.0 | 53.0 – 105.0 | U= 654.5 | 0.160 |
| Mean ± SD. | 73.6 ± 14.4 | 75.0 ± 9.4 |  |  |
| Median (IQR) | 70.0 (65.0 – 79.0) | 72.0 (70.0 – 80.0) |  |  |
| **SBP mmHg (day 2)** |  |  |  |  |
| Min. – Max. | 54.0 – 95.0 | 55.0 – 98.0 | U= 760 | 0.699 |
| Mean ± SD. | 74.5 ± 9.1 | 73.1 ± 9.7 |  |  |
| Median (IQR) | 72.5 (68.5 – 79.5) | 71.5 (70.0 – 79.0) |  |  |
| **SBP mmHg (day 3)** |  |  |  |  |
| Min. – Max. | 60.0 – 104.0 | 40.0 – 98.0 | t= 2.676 | 0.009^*^ |
| Mean ± SD. | 76.9 ± 9.7 | 70.7 ± 10.9 |  |  |
| Median (IQR) | 75.0 (70.0 – 81.0) | 70.0 (64.0 – 79.0) |  |  |
| **DBP mmHg (day 1)** |  |  |  |  |
| Min. – Max. | 23.0 – 63.0 | 16.0 – 63.0 | U= 618 | 0.079 |
| Mean ± SD. | 39.4 ± 9.5 | 41.9 ± 9.7 |  |  |
| Median (IQR) | 38.0 (32.5 – 45.0) | 42.5 (36.5 – 49.0) |  |  |
| **DBP mmHg (day 2)** |  |  |  |  |
| Min. – Max. | 21.0 – 60.0 | 23.0 – 60.0 | U= 698.5 | 0.328 |
| Mean ± SD. | 39.9 ± 8.7 | 41.6 ± 8.3 |  |  |
| Median (IQR) | 39.0 (35.0 – 44.0) | 42.0 (34.5 – 49.0) |  |  |
| **DBP mmHg (day 3)** |  |  |  |  |
| Min. – Max. | 30.0 – 75.0 | 23.0 – 64.0 | U= 656.5 | 0.167 |
| Mean ± SD. | 42.7 ± 8.9 | 39.8 ± 9.5 |  |  |
| Median (IQR) | 40.0 (36.5 – 49.0) | 39.0 (32.5 – 45.5) |  |  |
| **MBP mmHg (day 1)** |  |  |  |  |
| Min. – Max. | 31.0 – 85.0 | 30.0 – 80.0 | U= 569 | 0.026^*^ |
| Mean ± SD. | 51.1 ± 10.3 | 54.7 ± 9.2 |  |  |
| Median (IQR) | 50.0 (44.0 – 55.0) | 55.0 (48.5 – 59.0) |  |  |
| **MBP mmHg (day 2)** |  |  |  |  |
| Min. – Max. | 31.0 – 80.0 | 35.0 – 75.0 | U= 784 | 0.877 |
| Mean ± SD. | 52.4 ± 9.1 | 52.9 ± 8.0 |  |  |
| Median (IQR) | 51.5 (48.5 – 56.5) | 51.5 (47.0 – 58.0) |  |  |
| **MBP (day 3)** |  |  |  |  |
| Min. – Max. | 36.0 – 95.0 | 30.0 – 73.0 | U= 693.5 | 0.305 |
| Mean ± SD. | 53.5 ± 11.1 | 50.4 ± 9.0 |  |  |
| Median (IQR) | 52.5 (48.5 – 56.5) | 50.5 (45.0 – 55.5) |  |  |

U: Mann Whitney test t: Independent sample t test

p: p-value for comparing between the **two studied groups**

**S-Table (4): Comparison between the two groups as regards cardiovascular and respiratory support parameters**

|  | | **Group** | | **Test of sig.** | **P** |
| --- | --- | --- | --- | --- | --- |
|  |  | **NNG** | **Control** |  |  |
| **Need for Inotropes (day 1)** | Yes | 3 (7.5%) | 7 (17.5%) | χ^2^ = 1.829 | 0.176 |
|  | No | 37 (92.5%) | 33 (82.5%) |  |  |
| **Need for Inotropes (day 2)** | Yes | 6 (15.0%) | 16 (40.0%) | χ^2^ = 6.270 | 0.012^*^ |
|  | No | 34 (85.0%) | 24 (60.0%) |  |  |
| **Need for Inotropes (day 3)** | Yes | 8 (20.0%) | 25 (62.5%) | χ^2^ =14.907 | <0.001^*^ |
|  | No | 32 (80.0%) | 15 (37.5%) |  |  |
| **Surfactant** | Yes | 11 (27.5%) | 22 (55.0%) | χ^2^ = 6.241 | 0.012^*^ |
|  | No | 29 (72.5%) | 18 (45.0%) |  |  |

χ^2^: Chi-square test. p: p-value for comparing between the **two studied groups**

**S- table (5) table demonstrates the causes of PPHN in both studied groups.**

|  | | **Group** | | **Test** | **P** |
| --- | --- | --- | --- | --- | --- |
|  |  | **NNG** | **Control** |  |  |
| **Underlying cause** | **Primary** | **2** | **4** | **χ^2^ = 5.583** | **^MC^p = 0.238** |
|  |  | **5.0%** | **10.0%** |  |  |
|  | **Delayed transition with or without hypoxia** | **10** | **10** |  |  |
|  |  | **25.0%** | **25.0%** |  |  |
|  | **Secondary to pneumonia** | **8** | **15** |  |  |
|  |  | **20.0%** | **37.5%** |  |  |
|  | **Secondary to RDS** | **5** | **2** |  |  |
|  |  | **12.5%** | **5.0%** |  |  |
|  | **Secondary to meconium aspiration** | **15** | **9** |  |  |
|  |  | **37.5%** | **22.5%** |  |  |

**χ^2^: Chi-square test.**

**S-Table (6): Comparison between the two groups as regards outcome parameters**

|  | | Group | | **Test of sig.** | **P** |
| --- | --- | --- | --- | --- | --- |
|  |  | NNG | Control |  |  |
| **Duration of ventilation** | |  |  |  |  |
| Min. – Max. | | 2 – 24 | 2 – 26 | U= 463.5 | 0.001^*^ |
| Mean ± SD. | | 6 ± 4 | 9 ± 5 |  |  |
| Median (IQR) | | 5 (3 – 8) | 9 (5 – 12) |  |  |
| **Duration of hospitalization** | |  |  |  |  |
| Min. – Max. | | 5 – 25 | 4 – 39 | U= 493 | 0.003^*^ |
| Mean ± SD. | | 11 ± 5 | 16 ± 8 |  |  |
| Median (IQR) | | 10 (8 – 13) | 15 (10 – 20) |  |  |
| **Fate** | Died | 6 (15.0%) | 13 (32.5%) | χ^2^ = 3.382 | 0.066 |
|  | Discharged | 34 (85.0%) | 27 (67.5%) |  |  |

U: Mann Whitney test χ^2^: Chi-square test

p: p-value for comparing between the **two studied groups**

**s-Table (7): Life table demonstrating time course of weaning from ventilatory support (terminal events) and occurrence of death (withdrawn cases)**

| Group | Interval Start Time | Number Entering Interval | Number Withdrawing during Interval | Number of Terminal Events | Proportion Terminating | Proportion Surviving | Cumulative Proportion Surviving at End of Interval | Probability Density | Hazard Rate |
| --- | --- | --- | --- | --- | --- | --- | --- | --- | --- |
| **Case** | 0 | 40 | 0 | 3 | 0.08 | 0.93 | 0.93 | 0.001 | 0.00 |
|  | 50 | 37 | 0 | 15 | 0.41 | 0.59 | 0.55 | 0.008 | 0.01 |
|  | 100 | 22 | 0 | 10 | 0.45 | 0.55 | 0.30 | 0.005 | 0.01 |
|  | 150 | 12 | 0 | 5 | 0.42 | 0.58 | 0.17 | 0.003 | 0.01 |
|  | 200 | 7 | 3 | 1 | 0.18 | 0.82 | 0.14 | 0.001 | 0.00 |
|  | 250 | 3 | 0 | 0 | 0.00 | 1.00 | 0.14 | 0.00 | 0.00 |
|  | 300 | 3 | 1 | 0 | 0.00 | 1.00 | 0.14 | 0.00 | 0.00 |
|  | 350 | 2 | 1 | 0 | 0.00 | 1.00 | 0.14 | 0.00 | 0.00 |
|  | 400 | 1 | 0 | 0 | 0.00 | 1.00 | 0.14 | 0.00 | 0.00 |
|  | 450 | 1 | 0 | 0 | 0.00 | 1.00 | 0.14 | 0.00 | 0.00 |
|  | 500 | 1 | 0 | 0 | 0.00 | 1.00 | 0.14 | 0.00 | 0.00 |
|  | 550 | 1 | 1 | 0 | 0.00 | 1.00 | 0.14 | 0.00 | 0.00 |
| **Control** | 0 | 40 | 0 | 2 | 0.05 | 0.95 | 0.95 | 0.001 | 0.00 |
|  | 50 | 38 | 2 | 3 | 0.08 | 0.92 | 0.87 | 0.002 | 0.00 |
|  | 100 | 33 | 3 | 6 | 0.19 | 0.81 | 0.71 | 0.003 | 0.00 |
|  | 150 | 24 | 0 | 4 | 0.17 | 0.83 | 0.59 | 0.002 | 0.00 |
|  | 200 | 20 | 2 | 7 | 0.37 | 0.63 | 0.37 | 0.004 | 0.01 |
|  | 250 | 11 | 1 | 2 | 0.19 | 0.81 | 0.30 | 0.001 | 0.00 |
|  | 300 | 8 | 0 | 3 | 0.38 | 0.63 | 0.19 | 0.002 | 0.01 |
|  | 350 | 5 | 2 | 0 | 0.00 | 1.00 | 0.19 | 0.00 | 0.00 |
|  | 400 | 3 | 0 | 0 | 0.00 | 1.00 | 0.19 | 0.00 | 0.00 |
|  | 450 | 3 | 2 | 0 | 0.00 | 1.00 | 0.19 | 0.00 | 0.00 |
|  | 500 | 1 | 0 | 0 | 0.00 | 1.00 | 0.19 | 0.00 | 0.00 |
|  | 550 | 1 | 0 | 0 | 0.00 | 1.00 | 0.19 | 0.00 | 0.00 |
|  | 600 | 1 | 1 | 0 | 0.00 | 1.00 | 0.19 | 0.00 | 0.00 |

**s-Table (8): Life table demonstrating time course of weaning from ventilatory support (withdrawn cases) and occurrence of death (terminal events)**

| Group | Interval Start Time | Number Entering Interval | Number Withdrawing during Interval | Number of Terminal Events | Proportion Terminating | Proportion Surviving | Cumulative Proportion Surviving at End of Interval | Probability Density | Hazard Rate |
| --- | --- | --- | --- | --- | --- | --- | --- | --- | --- |
| **Case** | 0 | 40 | 3 | 0 | 0.0 | 1.0 | 1.0 | 0.0 | 0.0 |
|  | 50 | 37 | 15 | 0 | 0.0 | 1.0 | 1.0 | 0.0 | 0.0 |
|  | 100 | 22 | 10 | 0 | 0.0 | 1.0 | 1.0 | 0.0 | 0.0 |
|  | 150 | 12 | 5 | 0 | 0.0 | 1.0 | 1.0 | 0.0 | 0.0 |
|  | 200 | 7 | 1 | 3 | 0.46 | 0.54 | 0.54 | 0.009 | 0.01 |
|  | 250 | 3 | 0 | 0 | 0.0 | 1.0 | 0.54 | 0.0 | 0.0 |
|  | 300 | 3 | 0 | 1 | 0.33 | 0.67 | 0.36 | 0.004 | 0.01 |
|  | 350 | 2 | 0 | 1 | 0.5 | 0.5 | 0.18 | 0.004 | 0.01 |
|  | 400 | 1 | 0 | 0 | 0.0 | 1.0 | 0.18 | 0.0 | 0.0 |
|  | 450 | 1 | 0 | 0 | 0.0 | 1.0 | 0.18 | 0.0 | 0.0 |
|  | 500 | 1 | 0 | 0 | 0.0 | 1.0 | 0.18 | 0.0 | 0.0 |
|  | 550 | 1 | 0 | 1 | 1.0 | 0.0 | 0.0 | 0.004 | 0.04 |
| **Control** | 0 | 40 | 2 | 0 | 0.0 | 1.0 | 1.0 | 0.0 | 0.0 |
|  | 50 | 38 | 3 | 2 | 0.05 | 0.95 | 0.95 | 0.001 | 0.0 |
|  | 100 | 33 | 6 | 3 | 0.1 | 0.9 | 0.85 | 0.002 | 0.0 |
|  | 150 | 24 | 4 | 0 | 0.0 | 1.0 | 0.85 | 0.0 | 0.0 |
|  | 200 | 20 | 7 | 2 | 0.12 | 0.88 | 0.75 | 0.002 | 0.0 |
|  | 250 | 11 | 2 | 1 | 0.1 | 0.9 | 0.67 | 0.001 | 0.0 |
|  | 300 | 8 | 3 | 0 | 0.0 | 1.0 | 0.67 | 0.0 | 0.0 |
|  | 350 | 5 | 0 | 2 | 0.4 | 0.6 | 0.4 | 0.005 | 0.01 |
|  | 400 | 3 | 0 | 0 | 0.0 | 1.0 | 0.4 | 0.0 | 0.0 |
|  | 450 | 3 | 0 | 2 | 0.67 | 0.33 | 0.13 | 0.005 | 0.02 |
|  | 500 | 1 | 0 | 0 | 0.0 | 1.0 | 0.13 | 0.0 | 0.0 |
|  | 550 | 1 | 0 | 0 | 0.0 | 1.0 | 0.13 | 0.0 | 0.0 |
|  | 600 | 1 | 0 | 1 | 1.0 | 0.0 | 0.0 | 0.003 | 0.04 |

**S-Fig.(1)
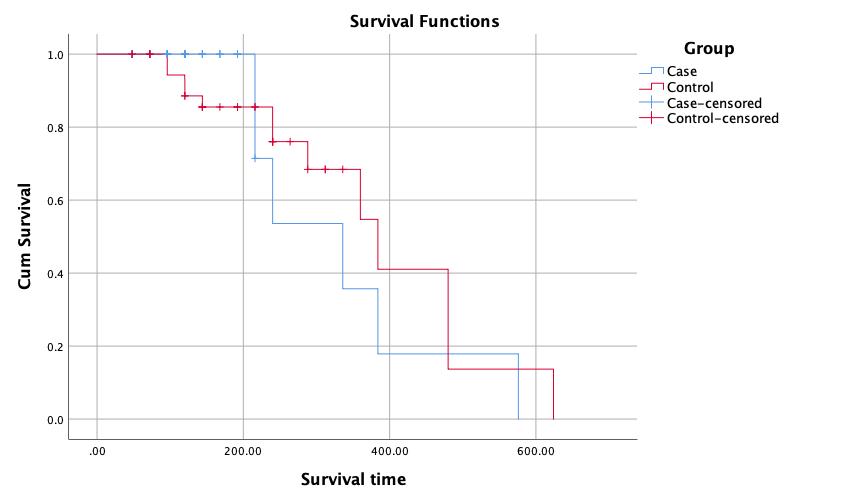
**

**Kaplan Meier survival plot representing time course of occurrence of death (terminal events) and weaning from ventilatory support (censored cases) in the two studied groups.**
