# Supplementary material for: Nebulized nitroglycerin as an adjuvant drug in management of persistent pulmonary hypertension of newborns: a randomized controlled trial
Source: Eur J Pediatr. 2025 Sep 1;184(9):586. doi: 10.1007/s00431-025-06381-5 (PMC12402028; doi:10.1007/s00431-025-06381-5)
Supplement: Supplementary file 2 — (DOCX 877 KB) [file 431_2025_6381_MOESM2_ESM.docx]

**e-Table (1): Comparison between the two groups as regards oxygenation and blood gas parameters, as well as MAP and modes of ventilation.**

|  | | **Group** | | **Test of sig.** | **P** |
| --- | --- | --- | --- | --- | --- |
|  |  | **NNG** | **Control** |  |  |
| **Oxygenation index (day 1)** | | **n= 40** | **n= 40** |  |  |
| Median (IQR) | | 8.19 (5.45 – 13.29) | 9.45 (4.75 – 12.95) | U=800 | 1.00 |
| **Oxygenation index (day 2)** | | **n= 37** | **n= 40** |  |  |
| Median (IQR) | | 7.58 (4.65 – 10.39) | 9.87 (6.69 – 13.68) | U=527 | **0.030*** |
| **Oxygenation index (day 3)** | | **n= 33** | **n= 36** |  |  |
| Median (IQR) | | 3.82 (2.87 – 6.57) | 15.43 (11.04 – 23.85) | U=176.50 | **<0.001*** |
| **Test of Sig.** | | χ^2Friedman^= 19.152 | χ^2Friedman^= 18.167 |  |  |
| **P** | | **<0.001*** | **<0.001*** |  |  |
| **Sig. between days** | | p1= 0.325 p2**< 0.001***, p3= **0.001*** | p1= 0.077 p2**< 0.001***, p3= **0.013*** |  |  |
| **Oxygen sat index (day 1)** | | **n= 40** | **n= 40** |  |  |
| Median (IQR) | | 4.55 (3.02 – 7.41) | 5.26 (2.63 – 7.22) | U= 800 | 1.00 |
| **Oxygen sat index (day 2)** | | **n= 37** | **n= 40** |  |  |
| Median (IQR) | | 4.21 (2.57 – 5.79) | 5.50 (3.71 – 7.63) | U= 527 | 0.03^*^ |
| **Oxygen sat index (day 3)** | | **n= 33** | **n= 36** |  |  |
| Median (IQR) | | 2.10 (1.57 – 3.65) | 8.61 (6.15 – 13.34) | U= 176.5 | <0.001^*^ |
| **Test of Sig.** | | χ^2Friedman^= 19.152 | χ^2Friedman^= 18.167 |  |  |
| **P** | | **<0.001*** | **<0.001*** |  |  |
| **Sig. between days** | | p1= 0.325 p2**< 0.001***, p3= **0.001*** | p1= 0.077 p2**< 0.001***, p3= **0.013*** |  |  |
| **PH (day 1)** | |  |  |  |  |
| Min. – Max. | | 7.18 – 7.51 | 7.12 – 7.44 | t= 0.531 | 0.597 |
| Mean ± SD. | | 7.34 ± 0.08 | 7.33 ± 0.07 |  |  |
| **PH (day 2)** | |  |  |  |  |
| Min. – Max. | | 7.11 – 7.58 | 7.12 – 7.56 | t= 0.176 | 0.861 |
| Mean ± SD. | | 7.33 ± 0.08 | 7.33 ± 0.07 |  |  |
| **PH (day 3)** | |  |  |  |  |
| Median (IQR) | | 7.38 (7.35 – 7.41) | 7.29 (7.25 – 7.37) | U= 437 | <0.001^*^ |
| **Test of Sig.** | | χ^2Friedman^= 6.792 | χ^2Friedman^= 4.353 |  |  |
| **P** | | **0.034*** | 0.113 |  |  |
| **Sig. between days** | | p1= 0.665 p2= **0.050***, p3= **0.016*** |  |  |  |
| **PCO2 mmHg (day 1)** | |  |  |  |  |
| Min. – Max. | | 21.0 – 64.0 | 18.9 – 65.4 | t= -0.377 | 0.707 |
| Mean ± SD. | | 40.1 ± 9.0 | 40.8 ± 7.9 |  |  |
| **PCO2 mmHg (day 2)** | |  |  |  |  |
| Median (IQR) | | 41.0 (35.0 – 50.0) | 38.5 (36.5 – 48.5) | U= 789.5 | 0.919 |
| **PCO2 mmHg (day 3)** | |  |  |  |  |
| Median (IQR) | | 41.0 (36.0 – 47.5) | 50.0 (39.5 – 54.5) | U= 512.5 | 0.006^*^ |
| **Test of Sig.** | | χ^2Friedman^= 2.981 | χ^2Friedman^= 15.208 |  |  |
| **P** | | 0.225 | **<0.001*** |  |  |
| **Sig. between days** | |  | p1= 0.737 p2< **0.001***, p3= **0.001*** |  |  |
| **HCO3 mmol/L (day 1)** | |  |  |  |  |
| Median (IQR) | | 22.2 (19.8 – 23.1) | 21.7 (18.8 – 23.0) | U= 738.5 | 0.553 |
| **HCO3 mmol/L (day 2)** | |  |  |  |  |
| Median (IQR) | | 22.4 (21.0 – 24.0) | 21.6 (19.1 – 23.0) | U= 601.5 | 0.056 |
| **HCO3 mmol/L (day 3)** | |  |  |  |  |
| Median (IQR) | | 22.5 (20.7 – 23.8) | 22.3 (19.0 – 24.0) | U= 679 | 0.244 |
| **Test of Sig.** | | χ^2Friedman^= 0.314 | χ^2Friedman^= 0.726 |  |  |
| **P** | | 0.855 | 0.696 |  |  |
| **MAPcmH2O (day 1)** | | **n= 40** | **n= 40** |  |  |
| Median (IQR) | | 10 (8 – 13) | 10 (7 – 12) | U= 685 | 0.265 |
| **MAP cmH2O (day 2)** | | **n= 37** | **n= 40** |  |  |
| Median (IQR) | | 10 (7 – 11) | 11.5 (8.25 – 12) | U= 549 | 0.05^*^ |
| **MAP cm H2O (day 3)** | | **n= 34** | **n= 36** |  |  |
| Median (IQR) | | 6 (5 – 10.25) | 13 (12 – 14) |  |  |
| **Test of Sig.** | | χ^2Friedman^= 20.045 | χ^2Friedman^= 23.044 |  |  |
| **P** | | **<0.001*** | **<0.001*** |  |  |
| **Sig. between days** | | p1= 0.102 p2**< 0.001***, p3= **0.006*** | p1= **0.039*** p2**< 0.001***, p3= **0.010*** |  |  |
| **Mode of ventilation (day 1)** | HFOV | 16 (40.0%) | 9 (22.5%) | χ^2^ = 3.190 | ^MC^p = 0.398 |
|  | PTV | 13 (32.5%) | 15 (37.5%) |  |  |
|  | NIPPV | 2 (5.0%) | 2 (5.0%) |  |  |
|  | NCPAP | 9 (22.5%) | 14 (35.0%) |  |  |
|  | HB | 0 (0.0%) | 0 (0.0%) |  |  |
|  | Free flow | 0 (0.0%) | 0 (0.0%) |  |  |
| **Mode of ventilation (day 2)** | HFOV | 12 (30.0%) | 11 (27.5%) | χ^2^ = 5.710 | ^MC^p = 0.349 |
|  | PTV | 16 (40.0%) | 16 (40.0%) |  |  |
|  | NIPPV | 1 (2.5%) | 5 (12.5%) |  |  |
|  | NCPAP | 8 (20.0%) | 8 (20.0%) |  |  |
|  | HB | 2 (5.0%) | 0 (0.0%) |  |  |
|  | Free flow | 1 (2.5%) | 0 (0.0%) |  |  |
| **Mode of ventilation (day 3)** | HFOV | 7^*<^ (17.5%) | 20^*>^ (50.0%) | χ^2^ = 35.993 | ^MC^p = <0.001^*^ |
|  | PTV | 1^*<^ (2.5%) | 12^*>^ (30.0%) |  |  |
|  | NIPPV | 1 (2.5%) | 2 (5.0%) |  |  |
|  | NCPAP | 25^*>^ (62.5%) | 2^*<^ (5.0%) |  |  |
|  | HB | 1 (2.5%) | 1 (2.5%) |  |  |
|  | Free flow | 5 (12.5%) | 3 (7.5%) |  |  |

U: Mann Whitney test t: Independent sample t test

p: p-value for comparing between the **two studied groups**

‘NNG nebulized nitroglycerine group

χ^2^: Chi-square test MC: Monte Carlo

p: p-value for comparing between the three studied groups           *: Statistically significant at p ≤ 0.05

*< significantly lower  *> significantly higher (post hoc analysis)

HFOV; high frequency oscillatory ventilation, PTV; patient triggered ventilation, NIPPV: nasal intermittent positive pressure ventilation, NCPAP: nasal continuous positive airway pressure, HB: head box

MAP: **Mean airway pressure**

p1; Significant between day 1 and day 2

p2; Significant between day 1 and day 3

p3; Significant between day 2 and day 3

**e-Table (2): Comparison between the two groups as regards echocardiographic parameters in the three scans.**

|  | | **Group** | | **Test of sig.** | **P** |
| --- | --- | --- | --- | --- | --- |
|  |  | **NNG** | **Control** |  |  |
| **sPAP (day 1) mmHg** | |  |  |  |  |
| Min. – Max. | | 25.0 – 85.0 | 20.0 – 65.0 | t= 2.527 | 0.014^*^ |
| Mean ± SD. | | 47.3 ± 13.4 | 40.2 ± 11.9 |  |  |
| **sPAP (day 2) mmHg** | |  |  |  |  |
| Median (IQR) | | 45 (35.0 – 50.0) | 40 (32.0 – 48.0) | U= 674 | 0.225 |
| **sPAP (day 3) mmHg** | |  |  |  |  |
| Median (IQR) | | 35 (29.0 – 40.75) | 47 (38.25 - 58.75) | U= 384.5 | <0.001^*^ |
| **Test of Sig.** | | χ^2Friedman^= 27.050 | χ^2Friedman^= 29.077 |  |  |
| **P** | | **<0.001*** | **<0.001*** |  |  |
| **Sig. between days** | | p1= 0.057 p2**< 0.001***, p3= **0.001*** | p1= 0.314 p2**< 0.001***, p3< **0.001*** |  |  |
| **sPAP/SSP (day 1)** | |  |  |  |  |
| Median (IQR | | 0.63 (0.48 – 0.85) | 0.50 (0.43 – 0.62) | U=560.5 | 0.021 * |
| **PDA (day 1)** | Yes | 33 (82.5%) | 31 (77.5%) | χ^2^ = 0.313 | 0.576 |
|  | No | 7 (17.5%) | 9 (22.5%) |  |  |
| **PDA (day 2)** | Yes | 33 (82.5%) | 29 (72.5%) | χ^2^ = 1.147 | 0.284 |
|  | No | 7 (17.5%) | 11 (27.5%) |  |  |
| **PDA (day 3)** | Yes | 27 (67.5%) | 27 (67.5%) | χ^2^ = 0 | 1.00 |
|  | No | 13 (32.5%) | 13 (32.5%) |  |  |
| **PDA size (day 1) mm** | |  |  |  |  |
| Median (IQR) | | 2.90 (2.18 – 3.30) | 3.00 (2.50 – 3.80) | U= 446 | 0.378 |
| **PDA size (day 2) mm** | |  |  |  |  |
| Median (IQR) | | 2.50 (2.04 – 3.10) | 3.00 (2.40 – 3.70) | U= 379 | 0.160 |
| **PDA size (day 3) mm** | |  |  |  |  |
| Median (IQR) | | 2.38 (1.89 – 3.00) | 3.00 (2.44 – 3.50) | U= 231 | 0.021^*^ |
| **Test of Sig.** | | χ^2Friedman^= 22.505 | χ^2Friedman^= 5.286 |  |  |
| **P** | | **<0.001*** | 0.071 |  |  |
| **Sig. between days** | | p1= 0.118 p2**< 0.001***, p3= **0.003*** |  |  |  |
| **PDA direction (day 1)** | Bidirectional | 22 (66.7%) | 18 (58.1%) | χ^2^ = 0.838 | ^MC^p =  0.622 |
|  | Left to Right | 8 (24.2%) | 8 (25.8%) |  |  |
|  | Right to Left | 3 (9.1%) | 5 (16.1%) |  |  |
| **PDA direction (day 2)** | Bidirectional | 15 (45.5%) | 14 (48.3%) | χ^2^ = 13.743 | 0.001^*^ |
|  | Left to Right | 17^*>^ (51.5%) | 5^*<^ (17.2%) |  |  |
|  | Right to Left | 1^*<^ (3.0%) | 10^*>^ (34.5%) |  |  |
| **PDA direction (day 3)** | Bidirectional | 3 (11.1%) | 6 (22.2%) | χ^2^ = 28.240 | ^MC^p = <0.001^*^ |
|  | Left to Right | 22^*>^ (81.5%) | 3^*<^ (11.1%) |  |  |
|  | Right to Left | 2^*<^ (7.4%) | 18^*>^ (66.7%) |  |  |
| **Ejection fraction (D1) (%)** | |  |  |  |  |
| Min. – Max. | | 45 – 88 | 38 – 86 | t= 0.674 | 0.502 |
| Mean ± SD. | | 69 ± 12 | 67 ± 11 |  |  |
| **Ejection fraction (D2) (%)** | |  |  |  |  |
| Min. – Max. | | 42 – 88 | 50 – 89 | t= 0.607 | 0.545 |
| Mean ± SD. | | 69 ± 10 | 68 ± 10 |  |  |
| **Ejection fraction (D3) (%)** | |  |  |  |  |
| Median (IQR) | | 72 (62 – 79) | 65 (57 – 72) | U= 505.5 | 0.005^*^ |
| **Test of Sig.** | | χ^2Friedman^= 0.731 | χ^2Friedman^= 5.592 |  |  |
| **P** | | 0.694 | 0.061 |  |  |
| **Fractional shortening (D1) (%)** | |  |  |  |  |
| Min. – Max. | | 21 – 55 | 17 – 51 | t= 0.815 | 0.418 |
| Mean ± SD. | | 36 ± 9 | 34 ± 8 |  |  |
| **Fractional shortening (D2) (%)** | |  |  |  |  |
| Median (IQR) | | 37 (33 – 41) | 34 (30 – 39) | U= 644.5 | 0.134 |
| **Fractional shortening (D3) (%)** | |  |  |  |  |
| Min. – Max. | | 20 – 51 | 19 – 49 | t= 2.393 | 0.019^*^ |
| Mean ± SD. | | 37 ± 8 | 33 ± 7 |  |  |
| **Test of Sig.** | | χ^2Friedman^= 0.247 | χ^2Friedman^= 4.397 |  |  |
| **P** | | 0.884 | 0.111 |  |  |
| **SVC VTI (day 1) (cm)** | |  |  |  |  |
| Min. – Max. | | 5.25 – 20.21 | 6.18 – 19.00 | t= 0.140 | 0.889 |
| Mean ± SD. | | 11.55 ± 3.67 | 11.43 ± 3.45 |  |  |
| **SVC VTI (day 2) (cm)** | |  |  |  |  |
| Median (IQR) | | 10.10 (8.55 – 13.50) | 11.30 (8.75 – 12.65) | U= 781.5 | 0.859 |
| **SVC VTI (day 3) (cm)** | |  |  |  |  |
| Median (IQR) | | 11.39 (9.20 – 14.00) | 12.00 (8.89 – 14.75) | U= 742.5 | 0.580 |
| **Test of Sig.** | | χ^2Friedman^= 4.608 | χ^2Friedman^= 2.850 |  |  |
| **P** | | 0.100 | 0.241 |  |  |
| **SVC diameter (day 1) (cm)** | |  |  |  |  |
| Median (IQR) | | 0.48 (0.41 – 0.50) | 0.50 (0.40 – 0.52) | U= 743 | 0.581 |
| **SVC diameter (day 2) (cm)** | |  |  |  |  |
| Median (IQR) | | 0.49 (0.41 – 0.53) | 0.49 (0.40 – 0.51) | U= 723.5 | 0.460 |
| **SVC diameter (day 3) (cm)** | |  |  |  |  |
| Median (IQR) | | 0.50 (0.43 – 0.53) | 0.50 (0.40 – 0.50) | U= 680 | 0.245 |
| **Test of Sig.** | | χ^2Friedman^= 1.238 | χ^2Friedman^= 1.568 |  |  |
| **P** | | 0.538 | 0.456 |  |  |
| **SVC flow (day 1) (ml‎/min)** | |  |  |  |  |
| Median (IQR) | | 285.0 (210.5 – 410.5) | 296.0 (187.0 – 398.0) | U= 799.5 | 0.996 |
| **SVC flow (day 2) (ml‎/min)** | |  |  |  |  |
| Median (IQR) | | 299.5 (178.8 – 426.5) | 279.5 (167.5 – 379.0) | U= 711.5 | 0.394 |
| **SVC flow (day 3) (ml‎/min)** | |  |  |  |  |
| Median (IQR) | | 312.5 (214.0 – 388.0) | 291.5 (215.5 – 391.5) | U= 769.5 | 0.769 |
| **Test of Sig.** | | χ^2Friedman^= 0.050 | χ^2Friedman^= 1.250 |  |  |
| **P** | | 0.975 | 0.535 |  |  |
| **SVC flow (day 1) (ml‎/min‎/kg)** | |  |  |  |  |
| Median (IQR) | | 108.39 (75.66 – 137.36) | 96.01 (68.53 – 132.34) | U= 728 | 0.488 |
| **SVC flow (day 2) (ml‎/min‎/kg)** | |  |  |  |  |
| Median (IQR) | | 115.96 (68.53 – 146.10) | 101.68 (67.99 – 116.45) | U= 643 | 0.131 |
| **SVC flow (day 3) (ml‎/min‎/kg)** | |  |  |  |  |
| Median (IQR) | | 114.26 (81.60 – 143.67) | 102.34 (72.38 – 141.09) | U= 716 | 0.419 |
| **Test of Sig.** | | χ^2Friedman^= 0.050 | χ^2Friedman^= 1.250 |  |  |
| **P** | | 0.975 | 0.535 |  |  |
| **TAPSE (day 1) (cm)** | |  |  |  |  |
| Median (IQR) | | 0.71 (0.60 – 0.80) | 0.75 (0.53 – 0.90) | U= 762.5 | 0.715 |
| **TAPSE (day 2) (cm)** | |  |  |  |  |
| Median (IQR) | | 0.80 (0.70 – 0.90) | 0.70 (0.60 – 0.90) | U= 679 | 0.239 |
| **TAPSE (day 3) (cm)** | |  |  |  |  |
| Median (IQR) | | 0.90 (0.76 – 1.08) | 0.70 (0.50 – 0.70) | U= 253 | <0.001^*^ |
| **Test of Sig.** | | χ^2Friedman^= 36.573 | χ^2Friedman^= 19.121 |  |  |
| **P** | | **<0.001*** | **<0.001*** |  |  |
| **Sig. between days** | | p1= **0.05*** p2**< 0.001***, p3< **0.001*** | p1= 0.576 p2**< 0.001***, p3= **0.001*** |  |  |
| **Right VTI (day 1) (cm)** | |  |  |  |  |
| Min. – Max. | | 3.8 – 17.60 | 3.00 – 17.10 | t= -0.256 | 0.799 |
| Mean ± SD. | | 9.13 ± 2.92 | 9.30 ± 3.28 |  |  |
| **Right VTI (day 2) (cm)** | |  |  |  |  |
| Median (IQR) | | 9.75 (7.72 – 11.48) | 9.80 (7.45 – 11.55) | U= 783 | 0.870 |
| **Right VTI (day 3) (cm)** | |  |  |  |  |
| Min. – Max. | | 6.40 – 16.40 | 3.70 – 17.10 | t= 2.195 | 0.031^*^ |
| Mean ± SD. | | 10.84 ± 2.56 | 9.52 ± 2.84 |  |  |
| **Test of Sig.** | | χ^2Friedman^= 15.248 | χ^2Friedman^= 1.950 |  |  |
| **P** | | **<0.001*** | 0.377 |  |  |
| **Sig. between days** | | p1= **0.004*** p2**< 0.001***, p3= 0.402 |  |  |  |
| **Pulmonary diameter (day 1) (cm)** | |  |  |  |  |
| Min. – Max. | | 0.30 – 1.05 | 0.45 – 1.11 | t= -3.040 | 0.003^*^ |
| Mean ± SD. | | 0.72 ± 0.17 | 0.83 ± 0.14 |  |  |
| **Pulmonary diameter (day 2) (cm)** | |  |  |  |  |
| Min. – Max. | | 0.30 – 1.10 | 0.56 – 1.14 | t= -0.584 | 0.561 |
| Mean ± SD. | | 0.78 ± 0.15 | 0.80 ± 0.14 |  |  |
| **Pulmonary diameter (day 3) (cm)** | |  |  |  |  |
| Min. – Max. | | 0.50 – 1.18 | 0.50 – 1.10 | t= 1.282 | 0.204 |
| Mean ± SD. | | 0.80 ± 0.14 | 0.76 ± 0.14 |  |  |
| **Test of Sig.** | | F= 5.765 | F= 4.110 |  |  |
| **P** | | **0.007*** | **0.024*** |  |  |
| **Sig. between days** | | p1= **0.002*** p2= **0.005***, p3= 0.330 | p1= 0.263 p2= **0.008***, p3= **0.035*** |  |  |
| **RVO (day 1) (ml‎/min‎/kg)** | |  |  |  |  |
| Median (IQR) | | 206 (128.5 – 279.25) | 229 (146.75 – 323.25) | U= 704 | 0.356 |
| **RVO (day 2) (ml‎/min‎/kg)** | |  |  |  |  |
| Median (IQR) | | 234.5 (195.25 – 320.5) | 210 (168.25 – 334.25) | U= 721.5 | 0.450 |
| **RVO (day 3) (ml‎/min‎/kg)** | |  |  |  |  |
| Median (IQR) | | 304 (226 – 382.5) | 189.5 (150.25 – 250.5) | U= 456.5 | 0.001^*^ |
| **Test of Sig.** | | χ^2Friedman^= 40.650 | χ^2Friedman^= 3.950 |  |  |
| **P** | | **<0.001*** | 0.139 |  |  |
| **Sig. between days** | | p1= **0.003*** p2**< 0.001***, p3= **0.001*** |  |  |  |
| **TD-MPI RV (day 1)** | |  |  |  |  |
| Min. – Max. | | 0.35 – 0.89 | 0.37 – 0.87 | t= 1.532 | 0.130 |
| Mean ± SD. | | 0.63 ± 0.12 | 0.59 ± 0.13 |  |  |
| **TD-MPI RV (day 2)** | |  |  |  |  |
| Min. – Max. | | 0.41 – 0.81 | 0.33 – 0.87 | t= -0.785 | 0.435 |
| Mean ± SD. | | 0.61 ± 0.11 | 0.63 ± 0.11 |  |  |
| **TD-MPI RV (day 3)** | |  |  |  |  |
| Median (IQR) | | 0.52 (0.44 – 0.62) | 0.60 (0.56 – 0.73) | U= 493.0 | 0.003* |
| **Test of Sig.** | | χ^2Friedman^= 12.050 | χ^2Friedman^= 15.832 |  |  |
| **P** | | **0.002*** | **<0.001*** |  |  |
| **Sig. between days** | | p1= 0.118 p2= **0.001***, p3= 0.057 | p1= **0.012*** p2**< 0.001***, p3= 0.180 |  |  |
| **Left VTI (day 1) (cm)** | |  |  |  |  |
| Median (IQR) | | 8.40 (7.32 – 10.15) | 8.00 (6.10 – 10.20) | U= 709.5 | 0.384 |
| **Left VTI (day 2) (cm)** | |  |  |  |  |
| Min. – Max. | | 5.10 – 15.20 | 3.50 – 12.20 | t= 2.429 | 0.017^*^ |
| Mean ± SD. | | 9.48 ± 2.53 | 8.20 ± 2.16 |  |  |
| **Left VTI (day 3) (cm)** | |  |  |  |  |
| Min. – Max. | | 3.90 – 15.30 | 4.50 – 15.00 | t= 2.383 | 0.02^*^ |
| Mean ± SD. | | 9.36 ± 2.56 | 8.07 ± 2.28 |  |  |
| **Test of Sig.** | | χ^2Friedman^= 3.862 | χ^2Friedman^= 1.070 |  |  |
| **P** | | 0.145 | 0.586 |  |  |
| **Aortic Diameter (day 1) (cm)** | |  |  |  |  |
| Median (IQR) | | 0.57 (0.51 – 0.61) | 0.58 (0.50 – 0.61) | U= 757 | 0.678 |
| **Aortic Diameter (day 2) (cm)** | |  |  |  |  |
| Median (IQR) | | 0.60 (0.53 – 0.64) | 0.57 (0.50 – 0.68) | U= 768 | 0.757 |
| **Aortic Diameter (day 3) (cm)** | |  |  |  |  |
| Median (IQR) | | 0.60 (0.51 – 0.68) | 0.55 (0.49 – 0.65) | U= 604.5 | 0.059 |
| **Test of Sig.** | | χ^2Friedman^= 10.931 | χ^2Friedman^= 4.204 |  |  |
| **P** | | **0.004*** | 0.122 |  |  |
| **Sig. between days** | | p1= 0.162 p2= **0.002***, p3= 0.083 |  |  |  |
| **LVO (day1) (ml‎/min‎/kg)** | |  |  |  |  |
| Median (IQR) | | 110 (88.25 – 147.25) | 102.5 (77.25 – 143.5) | U= 704.5 | 0.358 |
| **LVO (day2) (ml‎/min‎/kg)** | |  |  |  |  |
| Median (IQR) | | 123.5 (102 – 172) | 98 (68 – 144.5) | U= 527.5 | 0.009^*^ |
| **LVO (day3) (ml‎/min‎/kg)** | |  |  |  |  |
| Median (IQR) | | 131.5 (112.25 – 195.5) | 95 (66.25 – 140.25) | U= 443 | 0.001^*^ |
| **Test of Sig.** | | χ^2Friedman^= 8.541 | χ^2Friedman^= 1.532 |  |  |
| **P** | | **0.014*** | 0.465 |  |  |
| **Sig. between days** | | p1= 0.199 p2= **0.004***, p3= 0.105 |  |  |  |

sPAP: systolic Pulmonary artery pressure

U: Mann Whitney test t: Independent sample t test SVC: Superior vena cava

p: p-value for comparing between the **two studied groups**

RVO: Right ventricular output LVO: Left ventricular output

χ^2^: Chi-square test MC: Monte Carlo

p: p-value for comparing between the three studied groups           *: Statistically significant at p ≤ 0.05

*< significantly lower  *> significantly higher (post hoc analysis)

PDA: Patent ductus arteriosus PAP pulmonary artery pressure

**eFig. 1: graphical presentations of clinical, oxygenation, laboratory and echocardiographic data in the studied groups**

**eFigure 2a: Vital signs comparison between NNG and control group.**


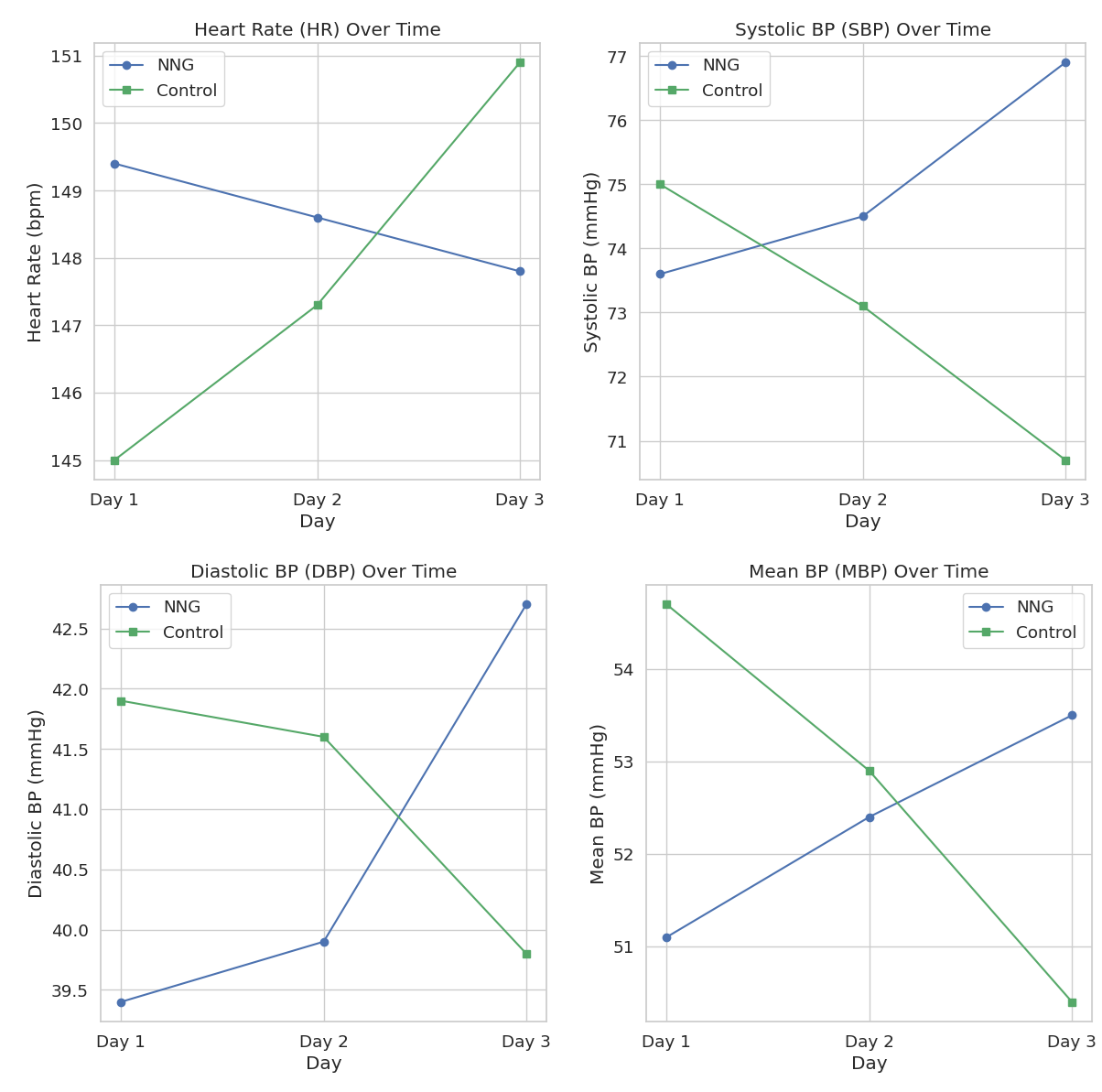


**P = 0.009**

**clinica**

**P = 0.026**

HR: beat/min, blood pressure(systolic diastolic and mean): mmHg

**eFigure 2b: Blood gas comparison between NNG and control group.**

**
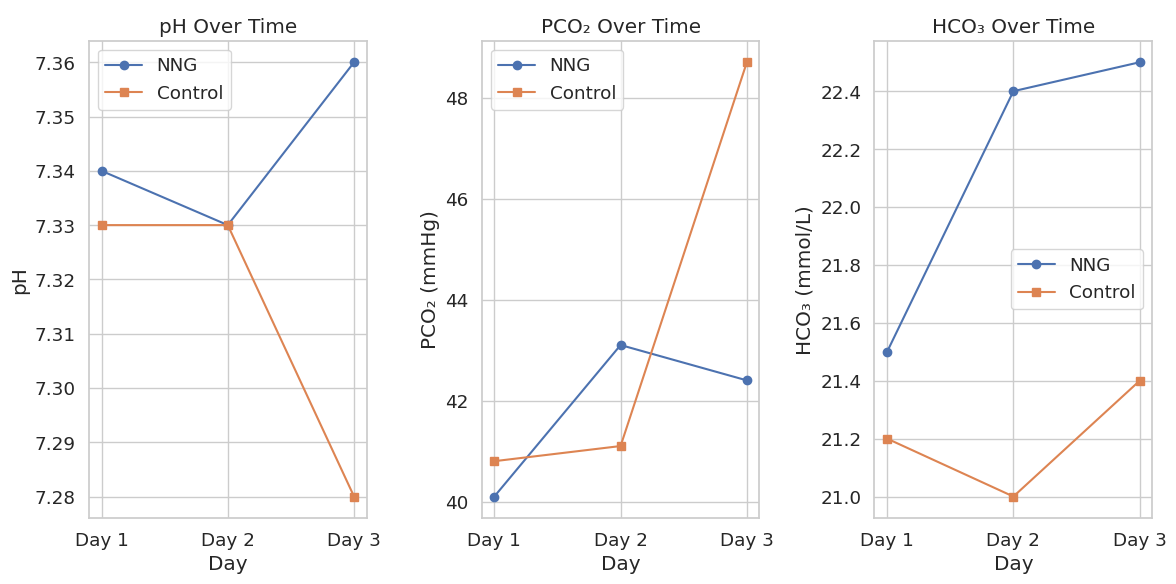
**

**P = 0.006**

**P = <0.001**


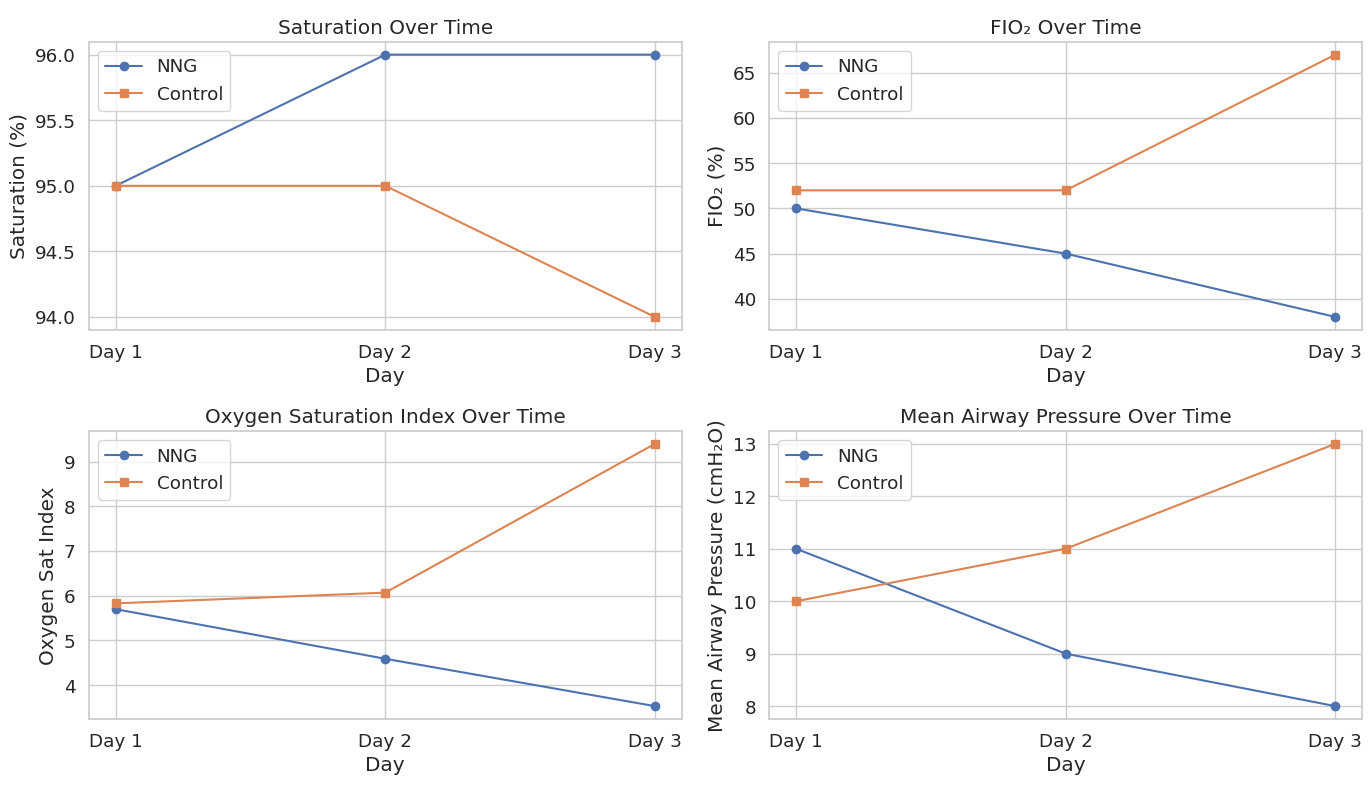
**eFigure (2c):** **Saturation Parameters comparison between NNG and control group**

**P = <0.001**

**P = 0.026**

**P = 0.039**

**P = <0.001**

**P = <0.001**

**P = 0.05**

**P = 0.03**


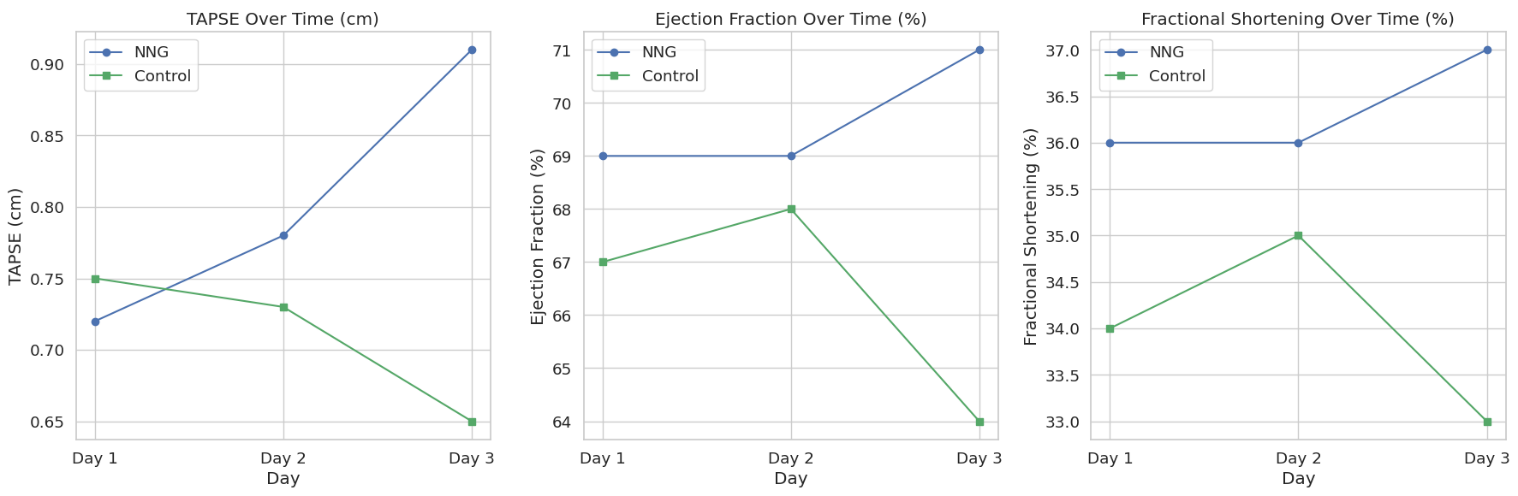
**eFigure (2d): TAPSE, EF, and FS comparison between NNG and control group.**

**P = 0.019**

**P = 0.005**

**P = <0.001**

**eFigure (2e**): **RVO parameters comparison between NNG and control group.**


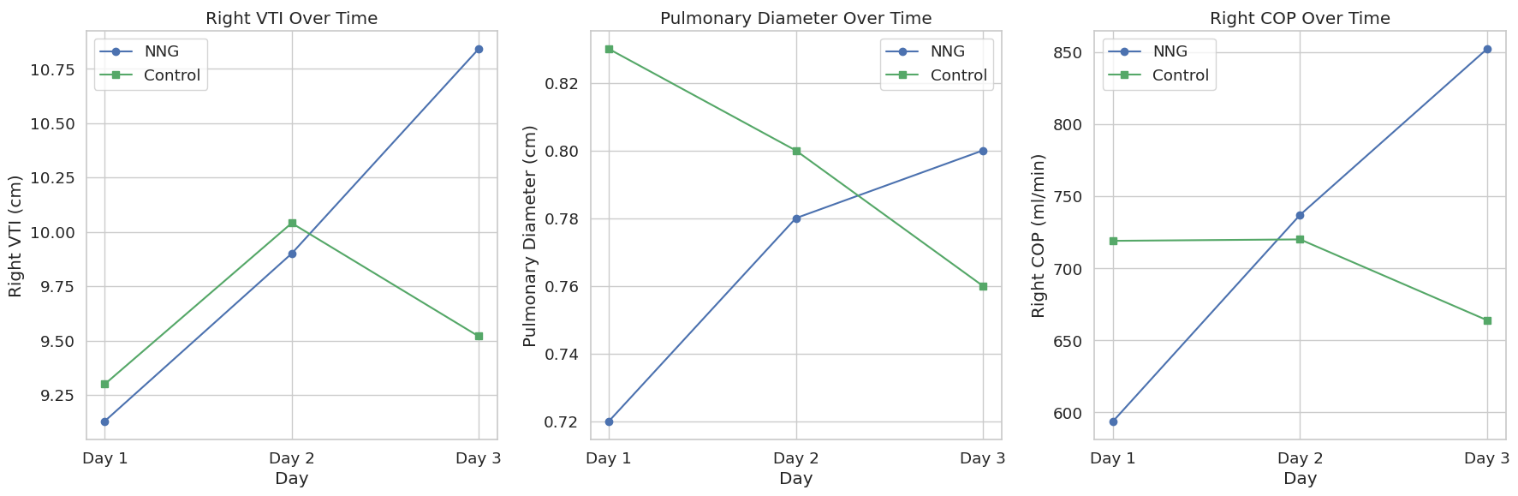


**P = 0.006**

**P = 0.003**

**P = 0.031**

VTI and pulmonary artery diameters: cm, RVO=Right COP: ml/kg /min

**eFigure (2f):** LVO parameters comparison between NNG and control group.


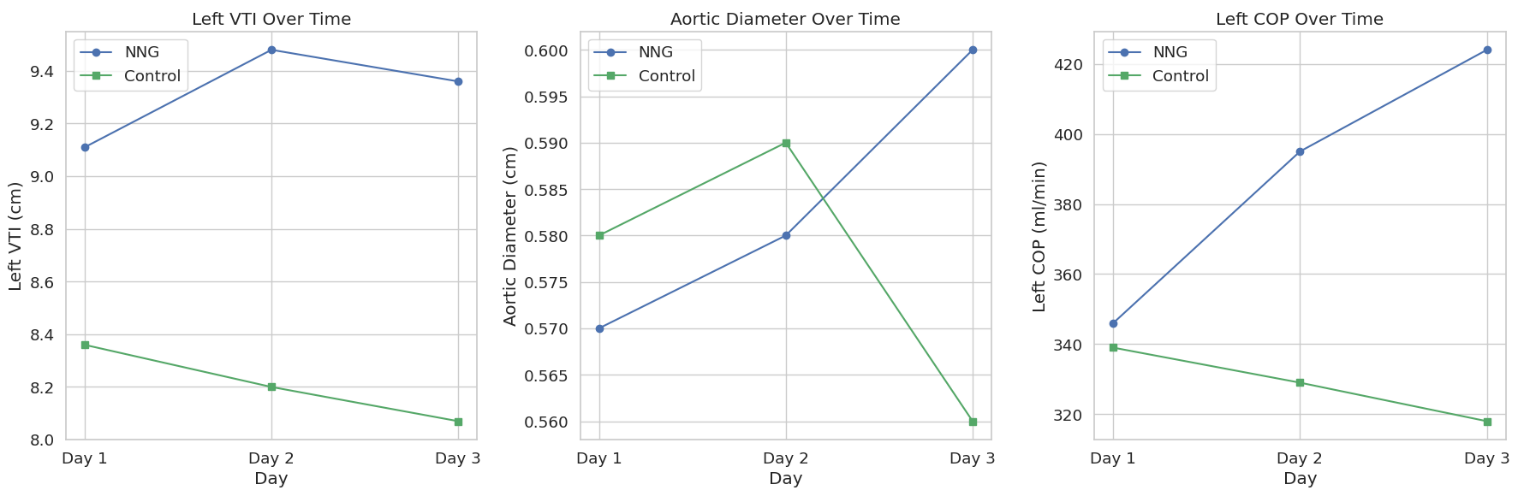


**P = 0.004**

**P = 0.017**

**P = 0.048**

**P = 0.02**

VTI and Aortic artery diameters: cm, LVO=left COP: ml/kg /min

**eFigure (2g): Ventricular function parameters comparison between NNG and control group.**


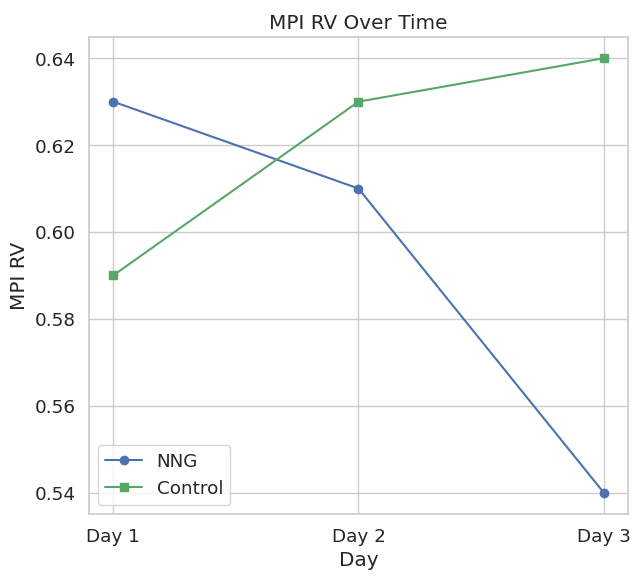


**P = 0.003**

**eFigure (2h):** **PDA size comparison between NNG and control group.**


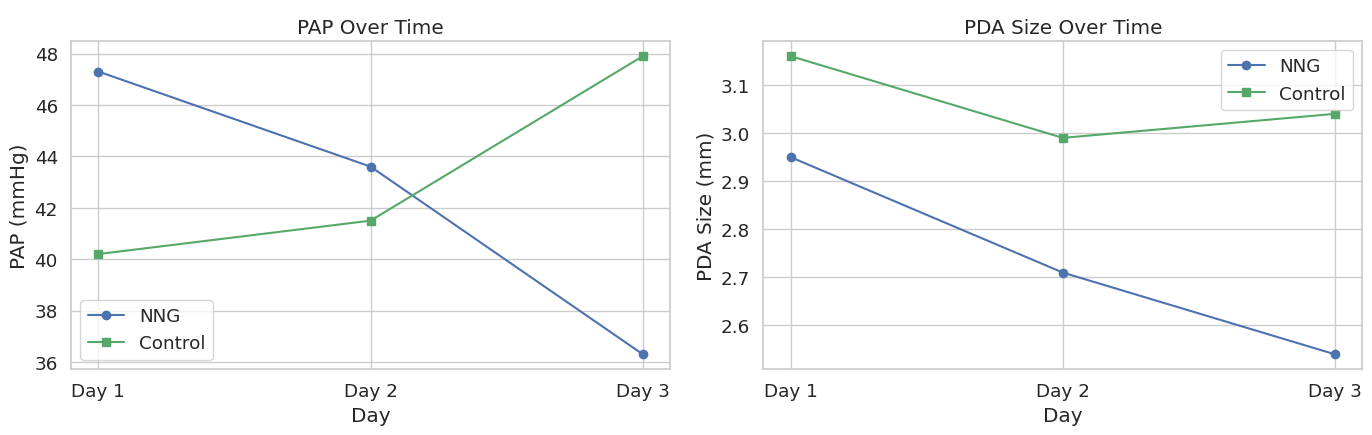


**P = 0.014**

**P = <0.001**

**P = 0.021**

PDA size: mm, PAP: mmHg
